# Involvement of 5-Serotonin and Substance *p* Pathways in Dichroa Alkali Salt-Induced Acute Pica in Rats

**DOI:** 10.3389/fphar.2021.588837

**Published:** 2021-04-22

**Authors:** Lina Ma, Sidi Li, Jian Li, Guangping Zhang, Hongping Hou, Zuguang Ye

**Affiliations:** ^1^ Institute of Chinese Materia Medica, China Academy of Chinese Medical Sciences, Beijing, China; ^2^ Post-doctoral Scientific Research Center, China Academy of Chinese Medical Sciences, Beijing, China

**Keywords:** dichroa alkali salt, pica in rats, emesis, 5-Serotonin, substance P

## Abstract

Dichroa alkali salt (DAS) is the active ingredient of Changshan, a traditional Chinese antimalarial medicine. However, owing to its vomiting side effects, its clinical use is limited. Recently, DAS-induced vomiting has attracted broad attention; however, the mechanisms involved have not yet been elucidated. The present study aimed to explore DAS induced vomiting and decipher the potential role of the 5-serotonin (5-HT) and substance *p* (SP) signaling pathways. We used a combination of approaches in the context of a rat pica model, such as immunoblot analysis, HPLC-ECD, ELISA, quantitative real-time PCR, pharmacological inhibition, and immunohistochemistry assays. We demonstrated that DAS contributed to Changshan-induced vomiting via the activation of the 5-HT and SP signaling pathways. DAS could induce a dose-dependent kaolin intake in the rat pica model. Moreover, DAS caused a similar profile as Cisplatin (DDP): “low-dose double-peak, high-dose single-peak pica phenomenon”. Interestingly, treatment with DAS stimulated the peripheral ileum and central medulla oblongata and augmented the release of 5-HT, SP, and preprotachykinin-A and the expression of 5-HT_3_ and NK_1_ receptors in the two issues in acute phase. Additionally, the 5-HT_3_ and NK_1_ receptor antagonists effectively alleviated DAS-induced kaolin intake and significantly reduced DAS-induced 5-HT and SP levels in the two issues in acute phase. Similar responses were not observed in the context of dopamine receptor inhibition. This study innovatively revealed that the 5-HT and SP-mediated vomiting network plays an important role in DAS-induced acute vomiting; of note, ondansetron, and aprepitant can effectively antagonize DAS-induced vomiting. Our results suggest a potential therapeutic strategy (based on drugs approved for human use) to prevent the DAS-associated adverse reactions.

## Introduction

Malaria is a vector-borne infectious disease caused by a few species of *Plasmodium* parasites, transmitted by the bite of *Anopheles* mosquitoes. It is one of the most important public health issues, globally. Nearly 250 million people worldwide contract malaria each year, and 860,000 people die, of which approximately 85% are children (WHO, 2018). The traditional treatments for malaria are chloroquine or quinine, but their efficacy is decreasing due to the emergence of drug resistance. At the end of the 1960s, efforts to eradicate malaria suffered setbacks, and its incidence started to increase on a year by year basis. Thus, malaria has attracted great attention from various countries competing to develop new anti-malarial drugs. Tu Youyou, a Chinese scientist, first discovered artemisinin from a treasure trove of traditional Chinese medicine (Collaboration Research Group for Qinghaosu, 1977); of note, artemisinin showed a significant effect on chloroquine-*resistant Plasmodium* parasites. Indeed, Chinese medicine has helped to control the global malaria epidemic and has saved at least 100,000 people every year. In recognition of Tu Youyou’s achievements with respect to malaria treatment, the Swedish Caroline School of Medicine awarded her the Nobel Prize in Physiology or Medicine (Tu, 2016). To prevent the emergence of artemisinin drug resistance, the WHO has repeatedly reminded the public health departments in all countries to use drugs cautiously and promoted the use of anti-malaria combination therapies (artemisinin combination therapies, ACTs) (WHO, 2006). However, in recent years, with the widespread use of artemisinin in malaria-endemic regions around the world, artemisinin-resistant parasites have inevitably appeared. In particular, the “Super Malaria” discovered in Cambodia in 2007 has now spread to Thailand, Laos, and Myanmar (Dondorp et al., 2009), and there is also a “Super Malaria” epidemic in Vietnam. Of note, in Vietnam, resistance to artemisinin-based treatments already accounts for a third of all malaria cases (Ashley et al., 2014. Michael Chew of the Wellcome Trust said: “The spread of this malaria superbug, resistant to the most effective drugs we have, is worrying and has a major impact on public health”. From quinine to chloroquine to today’s clinical first-line drugs, drug resistance is an unavoidable problem in the struggle between humans and malaria. Although ACT is still the most effective way to treat malaria worldwide, the emergence of artemisinin resistance requires counteractive measures. Tu Youyou, the inventor of artemisinin, believes that the replacement of adjuvant drugs that are no longer effective in the context of artemisinin combination therapy can effectively improve the treatment of malaria patients (Wang et al., 2019). Therefore, there is an urgent need to develop new antimalarial drugs with very different structures to cope with a possible global malaria outbreak caused by widespread drug resistance.

Changshan is the dry root of *Hydrangea febrifuga* (Lour.) Y. De Smet and Granados, a traditional Chinese medicine (TCM) used for the treatment of malaria and phlegm, whose properties, based on the TCM theory, are bitter, pungent, and cold. Traditionally, Changshan is used in the treatment of malaria of various etiologies, such as malaria caused by *Plasmodium vivax*, and *P. falciparum*, and intractable malaria, but also other diseases, such as chicken coccidiosis, Giardia lambliasis, and other parasitic diseases. In fact, previous studies showed that Changshan and its active ingredients have various pharmacological effects: anti-amoeba, anti-leptospira, anti-viral, anti-tumor, anti-hypertensive emetic, and uterine smooth muscle stimulator (Jiang et al., 2005; Figueiredo-Pontes et al., 2007; Cook et al., 2010; Pandey et al., 2017) (Henderson and Rose, 1949). Modern pharmacological and chemical studies have shown that the main active ingredients of Changshan are febrifugine and isofebrifugine (Jang and Fu, 1946; Koepfli et al., 1947; Chou and Fu, 1948). They are a class of new quinazoline compounds with a structure completely different from the artemisinin peroxygen bridge structure. The famous pharmacologist Professor C. S. Jang and his collaborators first discovered and isolated the effective alkaloids of Changshan with anti-malaria properties, and systematically studied their clinical antimalarial activity and vomiting side effects (Zhou and Jang, 1943; Jang and Zhou, 1955; Chiang et al., 1957; Chiang, 1961). Subsequently, using samples from Dr T. Q. Chao, the Director of the Institute Materia Medica, Shanghai, the American scholar K. K. Chen also studied their antimalarial activity on malaria models using ducks, canaries, and monkeys and found that it was 100 times greater than that of quinine. However, the intractable nausea (and vomiting) caused by febrifugine has limited its therapeutic use and prevented its popularization and application.

In fact, since ancient times, vomiting is the most common clinical adverse reaction associated with Changshan. Many researchers have attempted to eliminate the vomiting side effects caused by Changshan through structural modification and derivatization; however, these attempts have not been successful. Professor C.S. Jang, who discovered dichroine, used a pigeon and a surgical dog model, and found that destroying the dog’s vomiting center had no obvious effect on dichroine-induced vomiting. On the other hand, when all gastrointestinal vagus nerves were cut, dichroine did not induce vomiting using the threshold or large doses, suggesting that the emetic effect of dichroine occurs mainly through the reflex caused by the stimulation of the gastrointestinal vagus nerve (Chiang et al., 1957; Chiang, 1961). Some scholars believe that the effect of dichroine on pigeons is central, but experimental evidence is inconclusive (Henderson and Rose, 1949). Thus, it remains unclear whether the vomiting induced by dichroine is caused by direct peripheral stimulation or via action on the chemoreceptor trigger zone (CTZ) vomiting center. In a preliminary study, we found that dichroine is unstable and easily undergoes isomeric changes in aqueous solution. Subsequently, in the context of structural optimization, we observed that dichroa alkali salt (DAS) test samples were more stable and showed a controllable quality and a considerable purity, which ensured the smooth conduction of subsequent experiments. Therefore, based on our previous work, this study aims to systematically explore the characteristics and mechanism of DAS-induced vomiting.

Recent studies showed that chemical drugs can act directly on the intestinal mucosa or via blood circulation to induce enterochromaffin cells (EC) to release neurotransmitters, which activate receptors on the abdominal vagus afferent nerve terminals. This causes neurotransmission to the vomiting center and vomiting (Rojas and Slusher, 2012). There are abundant neurotransmitter receptors in the vagus dorsal nucleus complex (located in the medulla oblongata) that may play an important role in the vomiting mechanism. For instance, neurokinin-1 (NK_1_) receptors (Saito et al., 2003; Schank and Heilig, 2017) 5-serotonin 3 (5-HT_3_) receptors (Gan, 2005; Fakhfouri et al., 2015), dopamine (DA) receptors (Fortin et al., 2016), when in contact with substance *p* (SP), 5-HT and DA, respectively, may stimulate the vagus nerve, and eventually act on the vomiting center. In addition, these neurotransmitters can also directly act on central neurotransmitter receptors to directly promote vomiting. Based on the corresponding mechanism, antiemetics were developed, such as aprepitant (Apr, an NK_1_ receptor antagonist), ondansetron (Ond, a 5-HT_3_ receptor antagonist), and metoclopramide (Met, a DA receptor antagonist). Combining test drugs with antagonists whose mechanism of action is clear is an important means to explore the vomiting mechanism of emetogenic drugs whose mechanism of action is unclear. Therefore, in this study, we focused on the use of three well-known representative antiemetic drugs in combination with DAS to discover how DAS promotes vomiting.

Nowadays, there are many animal models used in preclinical vomiting research. The traditional models are dogs, cats, and pigeons. However, because dogs and cats quickly learn that a drug induces vomiting, vomiting may occur even if no emetic agent is given, which may lead to less rigorous studies. Moreover, due to animal protection laws, high price, and operational unsuitability, these models are rarely used (Chiang, 1961). The anatomical structure of pigeons is far different from that of mammals (Chiang et al., 1957), and pigeons do not have the same chemoreceptive region in the medulla oblongata as dogs and cats. The rat, one of the most commonly used laboratory animal species, cannot be used for direct assessment of emesis because most rodent species are unable to vomit. In the 1970s, the American scholar Mitchell et al. (Mitchell et al., 1976; Mitchell et al., 1977) first reported that emetic agents, such as cyclophosphamide, and motion sickness elicit dose-dependent pica in rats. Takeda et al. (Ossenkopp, 1983; Takeda et al., 1993) further showed that pica, an eating disorder that involves the satisfaction of a craving by eating kaolin, in rats may be analogous to gastrointestinal discomfort such as nausea and vomiting. Many experiments support the idea that pica in rats can be used as a good alternative rodent model to dogs and cats, particularly for preliminary and rapid screening of antiemetic agents (Ossenkopp, 1983; Takeda et al., 1995; Yamamoto et al., 2007). Further, pica in rats is believed to be mediated by the same mechanisms and possibly via the same receptors as vomiting in humans and other species possessing an emetic reflex (Takeda et al., 1993).

The aim of this study was to induce pica in rats and observe the effects of DAS (with or without Met, Ond, and Apr) on kaolin consumption (pica behavior). We focused on changes in vomiting-related neurotransmitters and their corresponding receptors in the peripheral ileum and central medulla oblongata, looking for antiemetic agents that could effectively counteract the vomiting effect induced by DAS. At the same time, we defined the role of different vomiting pathways in DAS-induced vomiting. Our results provide new insights for the prevention and treatment of DAS-induced emesis.

## Materials and Methods

### Animals

Male Sprague-Dawley rats (weighing about 200 g at the beginning of the experiment) were purchased from Vital River Laboratory Animal Technology, Co., Ltd (Beijing, China). Animals were kept under specific-pathogen-free conditions. All experiments were carried out in accordance with the recommendations of the ethical guidelines and regulations for the use of laboratory animals by the Institute of Chinese Materia Medica, China Academy of Chinese Medical Sciences, Beijing, China. All animal-related procedures adhered to the protocol approved by the Institutional Animal Care and Use Committee of the Institute of Chinese Materia Medica, China Academy of Chinese Medical Sciences.

### Reagents

Dichroa Alkali Salt (DAS) was isolated from D. febrifuga Lour (purity ≥99%), and dissolved in physiological saline. Cisplatin (DDP, Sigma-Aldrich, Beijing, China), metoclopramide hydrochloride (Met, Suicheng Pharmaceutical Co. Ltd. Xinzheng, China) and ondansetron hydrochloride (Ond, Qilu Pharmaceutical Co. Ltd.) were dissolved in physiological saline. Aprepitant (Apr), purchased from Merck Sharp and Dohme Ltd. (Hertfordshire, United Kingdom), was suspended in an aqueous suspension of 0.5% CMC (Sigma-Aldrich Japan). All drugs were prepared immediately before administration. 5-HT, 5-HIAA, gum arabic and kaolin were obtained from Sigma-Aldrich China. The SP ELISA kit was purchased from Cusabio Biotech Co. Ltd (Wuhan, Hubei, China). The anti-5-HT_3_ receptor antibody was obtained from Proteintech, Chicago, IL (cat. 10443-1-AP). The anti-NK_1_ receptor antibody was obtained from Santa Cruz Biotechnology, Inc (code sc-365091). Goat anti-rabbit IgG was obtained from the Proteintech Group (Chicago, IL, United States). Methanol (MeOH) and acetonitrile (ACN) were purchased from Fisher Chemicals (Hampton, NH, United States). All other substances (analytical grade), such as EDTA disodium, sodium 1-octanesulfonate, were purchased from the Beijing Chemical Factory (Beijing, China). The water for preparing the mobile phase was obtained using a Milli-Q system (Millipore, Madrid, Spain).

### Preparation of Kaolin Pellets

Kaolin pellets were prepared according to a previously reported method with a slight modification (Yamamoto et al., 2011). Briefly, pharmaceutical-grade kaolin (hydrated aluminum silicate) was mixed with 3% (w/w) gum arabic in distilled water to form pellets similar in size to chow pellets; these pellets were then completely dried at room temperature.

### Experimental Protocol and Drug Administration

**FIGURE 1:**
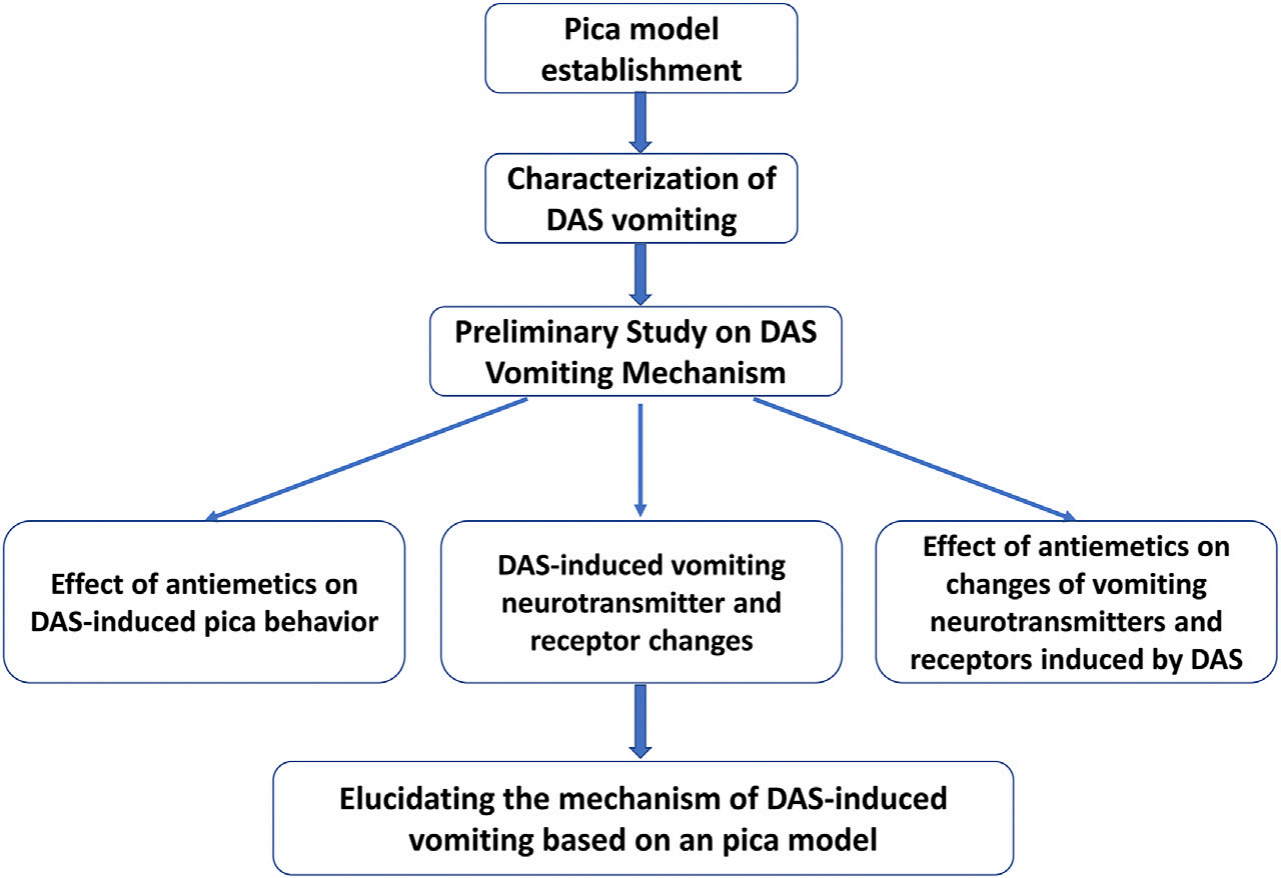
The flowchart of this study.

All rats were randomly divided into the experimental groups on a body weight-stratified basis at the end of the adaptation period. The experimental procedure is shown in Figure 1. First, using 5 ml/kg saline per os (p.o.) as a negative control and DDP 3 and 6 mg/kg (i.p.) in a volume of 5 ml/kg as a positive control, a rat pica model was established. Kaolin and food pellets were provided in their respective containers. Rats were adapted to the experimental environment for 3–5 days and allowed free access to tap water and both pellets throughout the experimental period. Kaolin intake, food intake, and body mass were measured to the nearest 0.01 g daily, between 8:00 and 9:00 a.m. for 4 days after administration. After the pica model was established, pica induced by DAS alone was characterized. Rats were administered different doses of DAS (2, 4, and 6 mg/kg, p. o.) in a volume of 5 ml/kg. Kaolin intake, food intake, and body mass were measured for 5 days. Afterward, the influence of different antiemetic agents on DAS-induced pica behavior was further observed to explore the possible emetic pathways of DAS. The experimental protocol was almost identical to the one used for the detection of DAS-induced pica behavior; the only difference was the administration of Met (2.1 and 4.2 mg/kg), Ond (0.83 and 1.66 mg/kg), and Apr (13.1 and 26.2 mg/kg) 30 min before the DAS administration. The clinical equivalent and double clinical equivalent doses were selected as the compatibility doses. After the emetogenic pathway induced by DAS was preliminarily identified via compatibility experiments, the effect of DAS on neurotransmitters and receptors in the related vomiting pathways was further investigated. At 0, 2, 4, 8, 24, 48, and 72 h after the administration of 2 mg/kg DAS, rats were deeply anesthetized via sevoflurane inhalation and pentobarbital. Their ileum and brains were removed, and the medulla oblongata was dissected from the surrounding tissues. The changes of 5-HT, 5-HIAA, and 5-HT_3_ receptor in the 5-HT_3_ receptor net in the ileum and medulla, and the expression of PPT-A, SP, and NK_1_ receptor in the NK_1_ receptor net of the two tissues was detected by the corresponding methods, respectively. Finally, we observed the effects of the 5-HT_3_ and NK_1_ receptor antagonists on DAS-induced changes in neurotransmitters and effector receptors in the two vomiting pathways to verify whether DAS could exert vomiting effects through these two pathways. Rats were administered Ond (1.66 mg/kg), Apr (26.2 mg/kg), or a combination 30 min before DAS (2 mg/kg, p. o.) to observe changes in neurotransmitters and effector receptor levels 4 h after administration.

### Assessment of 5-HT and 5-HIAA Using HPLC-ECD

Tissue samples were treated according to the method in the literature (Hu et al., 2004; Rojas et al., 2010): Briefly, frozen ileum or medulla tissue segments were weighed and homogenized in ice-cold 1.89% formic acid in water at a concentration of 10 ml/g tissue and centrifuged. ACN with 1% formic acid was added to the supernatant for protein precipitation (PPT) in a 3:1 proportion (v/v) and centrifuged at 14,000 rpm and 4°C for 5 min. Owing to the high concentration of the endogenous analytes (5-HT and 5-HIAA), the ileum or medulla tissue homogenates’ supernatants were diluted 100 times before PPT for the assays.

5-HT and 5-HIAA in the filtrates were measured by high-performance liquid chromatography (HPLC) (Sykam) with an electrochemical detector (Antec Decade ⅡSDC, Antec Scientific, Zoeterwoude, Netherlands). The mobile phase consisted of MeOH and water (10: 90, v/v) including 5 mg/L EDTA disodium, 190 mg/L sodium 1-octanesulfonate, and 17% MeOH. A glass carbon electrode, an *in-situ* silver/Ag/AgCl electrode (reference electrode), a 25 m vt-03 electrode spacer were used together with the Waters X Select @hss T3 chromatographic column (2.5 mm, 2.1 × 50 mm).

### Assessment of SP Levels Using an ELISA Commercial Kit

The obtained ileum and medulla homogenates were centrifuged at 3,000 rpm for 10 min and the supernatants were used to detect SP levels. SP concentrations were detected using an Rat Substance *p*, SP ELISA Kit (Cusabio Biotech Co. Ltd. Wuhan, Hubei, China, CSB-E08358), according to the manufacturer’s instructions.

### Quantitative Real-Time PCR Analysis

Total RNA was extracted from the rat ileum and medulla tissues using the total RNA kit (OMEGA, GA, United States) according to the manufacturer’s instructions. cDNA was obtained using the RevertAid First Strand cDNA Synthesis Kit (TRANSGEN BIOTECH, Beijing, China). The primers targeting *GAPDH*, *PPT-A*, *5-HT*_*3*_ receptor, and *NK*_*1*_ receptor were synthesized by the BGI (Shenzhen, China) and are listed in Table 1. The expression levels of these genes were examined via real-time PCR (Roche 480, Mannheim, Germany) using the Top Green qPCR SuperMix (TRANSGEN BIOTECH, Beijing, China). The dissociation curve analysis of the target genes showed a single peak. The real-time PCR conditions were as follows: 95°C for 5 min, followed by 40 cycles of 95°C for 10 s and 60°C for 10 s. The relative quantification of gene expression was calculated using the 2^−ΔΔCt^ method.

**TABLE 1:** List of primers (and the respective sequences) used for RT-PCR analysis in this study.

Primer		Sequence (5′to 3′)
NK_1_-R	Sense	AAACGCAAGGTGGTCAAA
	Antisense	GTCTGGAGGTATCGGGTG
5-HT_3_-R	Sense	AGCTGGTGCA TAAGCAGGATT
	Antisense	TCA​GTC​TTG​TTG​GCT​TGG​AAG​G
PPT-A	Sense	ACC​AGA​TCA​AGG​AGG​CAA​TG
	Antisense	GCC​CAT​TAG​TCC​AAC​AAA​GG
GAPDH	Sense	GCA​AGT​TCA​ACG​GCA​CAG​TCA
	Antisense	TGGTGGTGAAGA CGC CAGTAG

### Immunohistochemistry Assays

The primary antibodies used in this study were anti-5-HT_3_R (Proteintech, Chicago, IL, 10443-1-AP) and anti-NK_1_ receptor (Santa Cruz Biotechnology, Inc. sc-365091). Immunohistochemistry staining was performed using a commercial kit, according to the manufacturer’s instructions. 5-HT_3_ and NK_1_ receptor-positive signals were observed as brown or yellow granular masses in cells, and their intensities were measured using the ImageJ Analysis System (National Institutes of Health, Bethesda, MD, United States). A total of 15 fields per rat (three fields per section, five sections per rat, 400 × magnification) were randomly selected and analyzed. The positive staining intensity was calculated as the ratio of the stained area to the total fields assessed.

### Statistical Analysis

Data are expressed as the mean (M) ± standard error of the mean (SEM). Statistical comparisons were performed by two-way analysis of variance (ANOVA) and post-hoc test (ANOVA; factor 1 = treatment, factor 2 = time). Statistical analyses were performed using SPSS 20.0 software. Values of *p* < 0.05 were regarded as significantly different.

## Results

### Establishment of a Cisplatin-Induced Rat Pica Model

**FIGURE 2:**
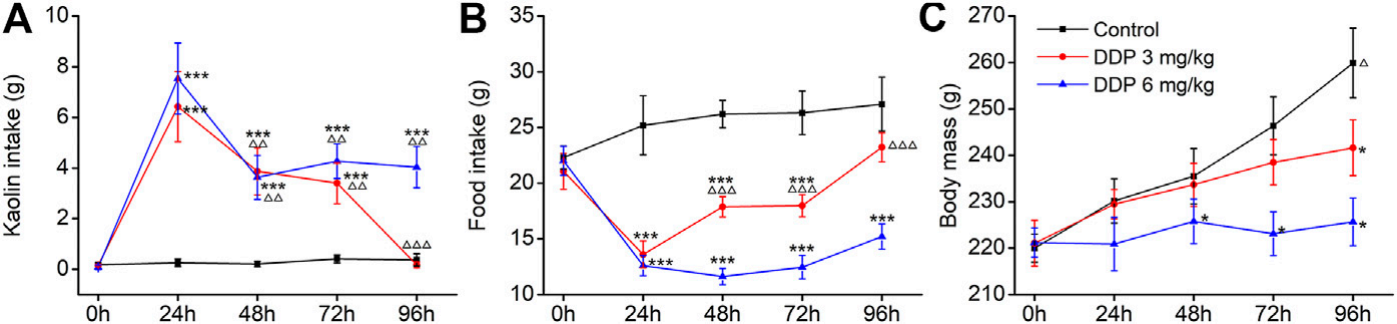
Effects of cisplatin (DDP) on kaolin intake **(A)** food intake, **(B)** and body mass, **(C)** in rats after a single administration (3 and 6 mg/kg, i. p.). Results of kaolin and food intake are shown as cumulative daily amounts (g) during 24 h periods up to 96 h after treatment. Body mass was weighed once per day. The data are expressed as mean ± SEM. Statistical significance was assessed using the one-way ANOVA. ^*^ *p* < 0.05, ^**^ *p* < 0.01, ^***^ *p* < 0.001 vs. the NC group, ^△^ *p* < 0.05, ^△△^ *p* < 0.01, ^△△△^ *p* < 0.001 vs. at 24 h.

The rat pica model was first established with Cisplatin (DDP; 3 and 6 mg/kg), used as the positive control. Rats consumed approximately 21.98–23.65 g food and approximately 0.03–0.96 g kaolin on the first day; however, little kaolin was eaten by rats during the subsequent 3–5 days. Therefore, all animals were acclimated to kaolin for 3–5 days before the execution of any experiments. As shown in Figures 2A,B, control animals (saline-treated) ate a small amount of kaolin (range 0–0.40 g) and approximately 25 g of food during the observation period. Importantly, we observed a significantly higher kaolin intake and reduced food intake in animals treated with DDP at days 1, 2 and 3 compared with those in the control group (*p* < 0.001), peaking on the first day. Compared with the first day, kaolin intake induced by 3 and 6 mg/kg DDP was significantly decreased at days 2–4 post-treatment (*p* < 0.01), suggesting that DDP-induced delayed-phase pica behavior was significantly weaker than that during the acute-phase. The pica behavior induced by 3 mg/kg DDP returned to normal at 96 h, whereas the pica behavior induced by 6 mg/kg DDP continued at 96 h. Although it was significantly lower than that of at 24 h after drug treatment (*p* < 0.01), the values were still significantly higher than in the blank group (*p* < 0.001). Further observation of the effect of DDP on normal food intake by rats showed that 3 and 6 mg/kg DDP significantly reduced the food intake by rats at 24, 48, 72, and 96 h after treatment. In contrast, food intake by rats in 3 mg/kg DDP group showed an increasing trend after 48 h, suggesting that the animal’s state gradually recovered, whereas food intake by rats in the 6 mg/kg DDP group did not improve significantly, indicating that DDP still played a role in gastrointestinal stimulation. As shown in Figure 2C, control rats generally gained approximately 10 g of body weight per day. Remarkably, 3 mg/kg DDP prevented this increase. This was evident at 72 and 96 h after treatment (*p* < 0.01). Moreover, rats injected with 6 mg/kg DDP lost weight, which lasted until 96 h after treatment (*p* < 0.01). This result was associated with marked suppression of food intake over a 3 day period. Since the pica behavior induced by high-dose DDP (6 mg/kg) was more obvious, lasted longer, and did not cause lethality during the observation period, this dose was selected for subsequent experiments.

### DAS-Induced Pica and Anorexia in Rats

**FIGURE 3:**
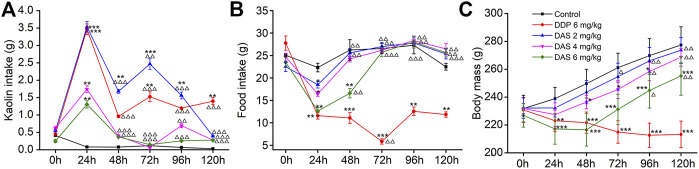
Effects of dichroa alkali salt (DAS) on kaolin intake **(A)** food intake, **(B)** and body mass, **(C)** in rats after a single administration. Control: saline, p. o.; DDP: 6 mg/kg, i. p.; DAS: 2, 4, and 6 mg/kg, p. o. Results of kaolin and food intake are shown as cumulative daily amounts (g) during 24 h periods up to 120 h after treatment. Body mass was weighed once per day. The data are expressed as mean ± SEM. Statistical significance was assessed using the one-way ANOVA. ^*^ *p* < 0.05, ^**^ *p* < 0.01, ^***^ *p* < 0.001 vs. the NC group, ^△^ *p* < 0.05, ^△△^ *p* < 0.01, ^△△△^ *p* < 0.001 vs. at 24 h.

Using saline as the negative control (NC) and 6 mg/kg DDP as the positive control, we investigated the sensitivity of the rat pica model to DAS. In the NC group, food intake was normal and no obvious kaolin intake was observed. The rats’ body weights increased continuously, and the animals were generally healthy. Compared with the NC group, the kaolin intake in the DDP-treated group was significantly increased 1 day (3.47 ± 0.17 g, *p* < 0.001) after treatment, while the food intake was significantly decreased (11.59 ± 0.76 g, *p* < 0.001). The body weight of the animals also decreased significantly, indicating the establishment of a successful rat pica model (Figure 3). Remarkably, different doses of DAS could also significantly induce pica behavior in rats, compared with the NC group. Surprisingly, the degree of DAS-induced pica was inversely correlated with the dose administrated: the higher the administered dose, the lower the kaolin intake. Indeed, rats treated with 2 mg/kg of DAS showed the most significant pica behavior (3.53 ± 0.15 g, *p* < 0.001); of note, these animals showed no less kaolin intake than the group treated with 6 mg/kg of DDP on the first day after treatment. On the following 2, 3, and 4 days, the kaolin intake of the DAS-treated groups was even higher than that of the DDP group, but on the fifth day after treatment (120 h post-DAS), the DAS-induced kaolin intake was similar to that of the NC group; of note the kaolin intake in DDP-treated animals on the fifth day remained at a relatively high level (1.09 ± 0.10 g, *p* < 0.01).

An interesting phenomenon in DAS-induced pica is that low-dose DAS (2 mg/kg) induced a biphasic kaolin intake profile, while higher DAS doses (4 and 6 mg/kg) showed a single peak profile. Therefore, the mechanism of different doses of DAS-induced pica behavior may be different. We found that 2 mg/kg DAS significantly increased kaolin intake at 24, 48, 72 and 96 h post-treatment (*p* < 0.01) and peaked after 24 h. The kaolin intake in the delayed phase was significantly lower than that in the acute phase (*p* < 0.001, *p* < 0.01, *p* < 0.001), reaching a peak 72 h post-treatment with 2 mg/kg DAS, and gradually decreasing during the next 48 h. 3.53 ± 0.15 g and 2.46 ± 0.16 g were the peak kaolin intake amounts observed during the acute and delayed phases, respectively. Overall, 9.63 + 0.47 g of kaolin were consumed during the entire 120 h period. On the other hand, the kaolin consumption in the high-dose DAS groups (4 and 6 mg/kg) only increased sharply in the first day, reaching a maximum (1.73 ± 0.10 and 1.30 ± 0.12, *p* < 0.01) after 24 h. Thereafter, kaolin consumption decreased to very low levels (less than 0.14 g per day); of note, the Kaolin intake was markedly lower compared to that of controls.

As shown in Figures 3B,C, the influence of DAS on food intake and body weight was overall positively correlated with the administered dose, indicating drug toxicity. Compared with the blank group, food intake in the DDP group decreased significantly on the first day after treatment and continued until the fifth day. The lowest food intake occurred on the third day after drug administration (5.84 ± 0.59, *p* < 0.01). However, the food intake of rats was reduced in a dose-dependent manner only on the first day after DAS treatment. We observed that food intake gradually recovered and returned to normal on the third day after drug administration (*p* > 0.05). On the first day after drug treatment, the food intake of animals in the 2 mg/kg DAS group (with the same kaolin intake of that in the DDP group) was much higher than that in animals treated with DDP (11.59 ± 0.76, 18.50 ± 0.75), suggesting that DAS and DPP induced pica via distinct mechanisms. Of note, although DAS could reduce rat body weight in a dose-dependent manner, with significant effects in the groups treated with 4 and 6 mg/kg doses, the body weight of the three DAS groups showed an increasing trend, while the body weight of the DDP group showed a downward trend. Compared with the NC group, the body weight of animals in the DAS group decreased in a dose-dependent manner during the observation period, with the highest reduction in the 6 mg/kg DAS group (*p* < 0.001), followed by the 4 mg/kg DAS group (*p* < 0.05), and then by the 2 mg/kg DAS group, the latest showing no significant difference compared to NC (*p* > 0.05). In summary, 2 mg/kg DAS induced clear pica behavior in rats, while minimally impacting food intake and body weight. Therefore, since this particular DAS dose did not affect the general animal state, it was used in the subsequent compatibility study.

### Effects of 5-HT_3_ and NK_1_ Receptor Antagonists on DAS-Induced Pica and Anorexia in Rats.

**FIGURE 4:**
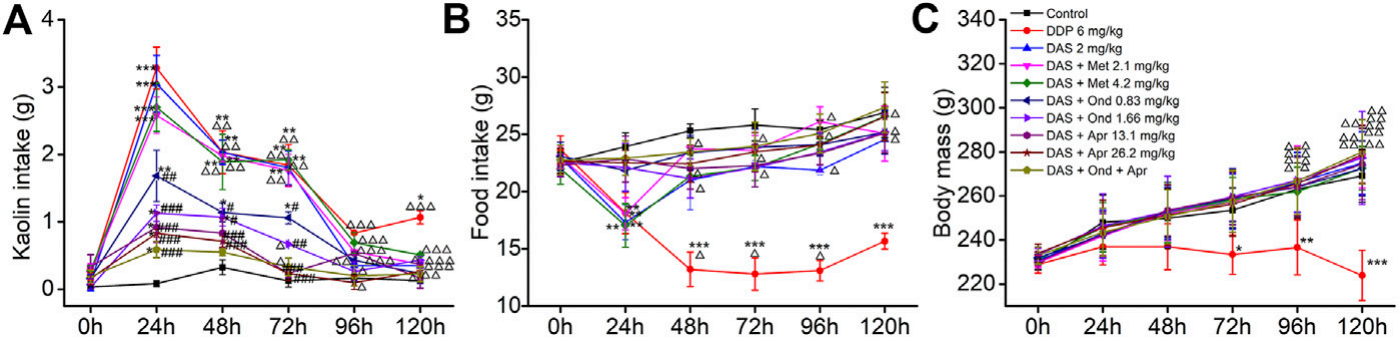
Effects of different antagonists on DAS-induced kaolin intake **(A)** food intake, **(B)** and body mass, **(C)** in rats. Control: saline, p. o.; DDP: 6 mg/kg, i. p.; DAS: 2 mg/kg, p. o.; and Metoclopramide: Met, 2.1 and 4.2 mg/kg, i. p.; Ondansetron: Ond, 0.83 and 1.66 mg/kg, p. o.; Aprepitant: Apr, 13.1 and 26.2 mg/kg p. o.; Met, Ond, and Apr were administered 30 min before DAS. The results of kaolin and food intake are shown as cumulative daily amounts (g) during 24 h periods up to 120 h after treatment. Body mass was weighed once per day. The data are expressed as mean ± SEM. Statistical significance was assessed using the one-way ANOVA. ^*^ *p* < 0.05, ^**^ *p* < 0.01, ^***^ *p* < 0.001 vs. the control group, and ^#^ *p* < 0.05, ^# #^ *p* < 0.01, ^# # #^ *p* < 0.001 vs. the DAS group, ^△^ *p* < 0.05, ^△△^ *p* < 0.01, ^△△△^ *p* < 0.001 vs. at 24 h.

Three antiemetic agents (Apr, Ond and Met) with clear anti-vomiting mechanisms were used to try to disclose the mechanism behind the DAS-induced pica behavior in rats. As shown in Figure 4A, the animals in the NC group ate a small amount of kaolin (range 0.03–0.16 g) and approximately 25 g food during the observation period after saline administration. Compared with the NC group, significantly increased kaolin intake and decreased food intake were observed in animals treated with DDP at 1, 2, 3, and 5 days. Of note, the highest amount of kaolin intake occurred on the first day after drug administration (3.28 ± 0.31, *p* < 0.001), suggesting once again that the rat pica model was successfully established. The kaolin intake of animals in the DAS group was similar to that of animals in the DDP group; still, the animals in the DDP group showed a significant kaolin intake on day 5 after treatment, while the animals in the DAS group showed no significant intake at the same time-point. After 1, 2, and 3 days of DAS administration, the kaolin intake was significantly increased, peaking on the first day post-DAS treatment (3.05 ± 0.42, *p* < 0.001). Interestingly, the 5-HT_3_ receptor antagonist Ond and the NK_1_-R antagonist Apr significantly reduced DAS-induced acute- and delayed-phase pica behavior (vs. DAS, *p* < 0.01), while the DA receptor antagonist Met had no significant effect (*p* > 0.05). These results suggest that the DA pathway does not play an obvious role in DAS-induced pica behavior. Of note, although Ond could dose-dependently inhibit DAS-induced rat pica on days 1, 2, and 3 post-treatment, it could not completely prevent DAS-induced pica (vs. control, *p* < 0.01), suggesting that the 5-HT_3_ receptor pathway is not the one solely responsible for DAS-induced vomiting. Similarly, although Apr could inhibit DAS-induced pica more strongly than Ond, it could not completely prevent pica, suggesting the NK_1_ receptor pathway may also play an important role in DAS-induced vomiting (but once again, not alone). Further observation of combined Ond and Apr treatment on DAS-induced pica behavior showed that although the combination could synergistically reduce DAS-induced pica, it still could not completely abrogate DAS-induced pica behavior.

We further observed the effect of different antiemetics on rat appetite and body weight (Figures 4B,C). Ond, Apr, and their combination effectively improved the reduction of food intake induced by DAS (vs. DAS, *p* < 0.01). However, Met did not improve the DAS-induced reduction of food intake (vs. DAS, *p* > 0.05). Since DAS only transiently reduces food intake, we did not observe significant changes in body weight (vs. control, *p* > 0.05). However, the food intake in the DDP group continued to significantly decrease (vs. control, *p* < 0.001), leading to a significant decrease in body weight, particularly relevant on the third day after DDP treatment (vs. control, *p* < 0.05).

### Effect of DAS on the Release of 5-HT and 5-Hydroxyindole Acetic Acid (5-HIAA), and the Expression of *5-HT*_*3*_ Receptor mRNA in the Ileum and Medulla of Rats.

**FIGURE 5:**
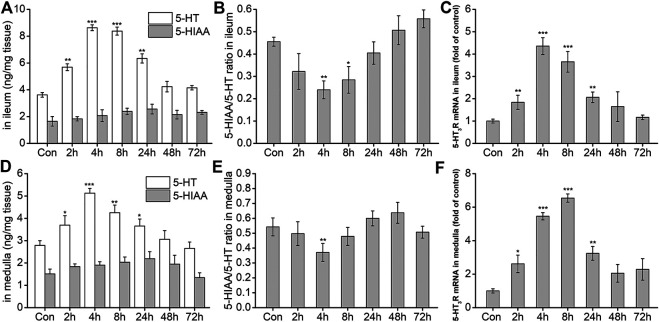
The effect of DAS on 5-HT and 5-HIAA secretion, and 5-HT3 receptor (5-HT3R) mRNA expression in the ileum and medulla of rats. Rats received DAS (2 mg/kg, p. o.), and their ileum and medulla were collected at 2, 4, 8, 24, 48, and 72 h after treatment (A, B, and C - ileum; D, E, and F - medulla). 5-HT and 5-HIAA concentrations were assessed using HPLC-ECD. The expression of *5-HT*_*3*_ receptor mRNA was detected using quantitative real-time PCR. The data are expressed as mean ± SEM. Statistical significance was assessed using the one-way ANOVA. ^*^ *p* < 0.05, ^**^ *p* < 0.01, ^***^ *p* < 0.001 vs. the control group.

After the possible pathways of DAS-induced vomiting were preliminarily identified using the rat pica surrogate model, we investigated changes in key neurotransmitters and related receptors in the two preliminarily identified pathways. The results are shown in Figure 5A. Compared with the NC group, 5-HT release in the peripheral ileum increased significantly at 2, 4, 8, and 24 h after DAS administration (*p* < 0.01), peaking at 4 h. No significant changes were observed at 48 and 72 h after drug administration (*p* > 0.05). 5-HTAA, a 5-HT metabolite, showed no significant changes at the different time-points investigated (*p* > 0.05). Next, the ratio of 5-HIAA to 5-HT was calculated (Figure 5B). The 5-HT metabolic rate decreased significantly from 2 h after treatment, reaching its minimum at 4 h, and gradually returning to normal at 24 h. Because the amount of 5-HTAA did not significantly change, our results suggest that DAS may increase its absolute content in the ileum by promoting 5-HT synthesis. Quantitative PCR was used to detect the expression of *5-HT*_*3*_ receptor mRNA in the ileum. We observed that *5-HT*_*3*_ receptor mRNA significantly increased at 2, 4, 8, and 24 h after DAS administration (vs. control, *p* < 0.01), also reaching a peak 4 h after drug administration (Figure 5C). Next, we investigated the secretion and expression of 5-HT and 5-HT_3_ receptor, respectively in the medulla oblongata. As shown in Figures 5D–F, 5-HT generation and decomposition in the medulla oblongata, and *5-HT*_*3*_ receptor gene expression were similar to those observed in the peripheral ileum; of note, 5-HT secretion in the medulla oblongata was slightly lower than that in the ileum.

### Effect of DAS on the Expression of *PPT-A* and *NK*_*1*_ *-R* mRNA, and SP Protein in the Ileum and Medulla of Rats.

**FIGURE 6:**
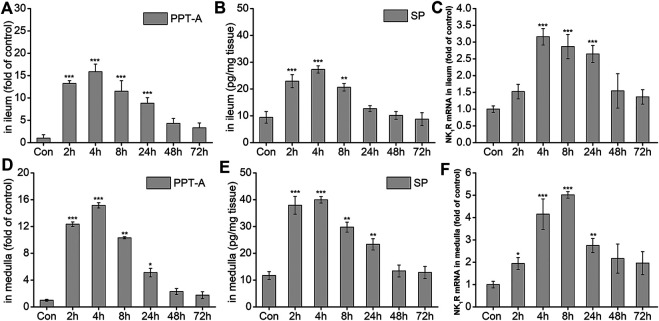
The effect of DAS on PPT-A mRNA, SP secretion, and NK1 receptor (NK1R) mRNA levels in the ileum and medulla of rats. Rats were received DAS (2 mg/kg, p. o.), and their ileum and medulla were collected at 2, 4, 8, 24, 48, and 72 h after treatment (A, B, and C - ileum; D, E, and F - medulla). *PPT-A* and *NK*_*1*_ *R* mRNA levels were detected using quantitative real-time PCR. S*p* concentration was detected using a commercial assay kit. The data are expressed as mean ± SEM. Statistical significance was assessed using the one-way ANOVA. ^*^ *p* < 0.05, ^**^ *p* < 0.01, ^***^ *p* < 0.001 vs. control the group.

The effects of DAS on the NK_1_ receptor pathway-related neurotransmitters and receptors were further investigated. As shown in Figure 6A, the expression of Preprotachykinin-A (*PPT-A*) mRNA in the ileum significantly increased at 2, 4, 8, and 24 h after drug treatment (*p* < 0.001), peaking at 4 h, and gradually returning to normal 48 h after treatment (*p* > 0.05). SP content in the ileum was similar to that of *PPT-A* mRNA at 2, 4, 8 h after treatment (*p* < 0.01, Figure 6B), returning to normal levels thereafter (*p* > 0.05). The expression of the SP-specific receptor *NK*_*1*_ *-R* mRNA in the ileum also increased significantly at 4, 8, and 24 h after DAS administration (*p* < 0.001), peaking at 4 h, and then gradually decreasing (Figure 6C). Further observation of SP and its receptor levels in the medulla oblongata showed that the overall changes in SP protein content and the expression of *PPT-A* and *NK*_*1*_ *-R* mRNA were similar to those in the ileum; of note, the SP content in the medulla oblongata was slightly higher than that in the ileum (Figures 6D–F).

### Effect of 5-HT_3_ or NK_1_ Receptor Antagonists on the DAS-Induced 5-HT and 5-HIAA Release in the Ileum and Medulla of Rats.

**FIGURE 7:**
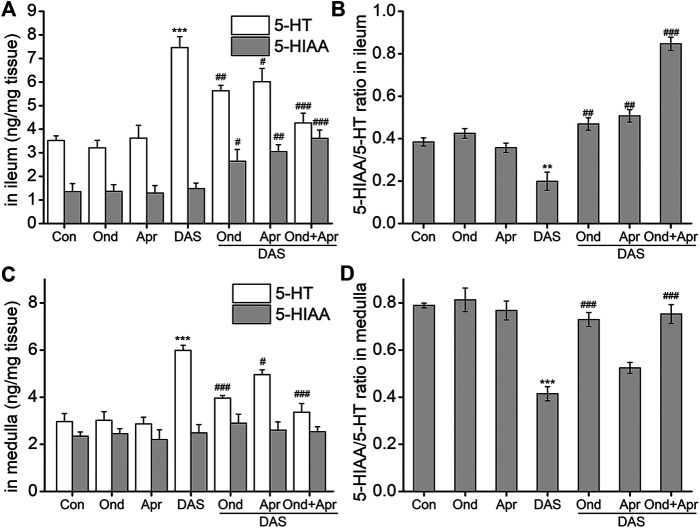
The effect of Ond and Apr on the DAS-induced 5-HT, and 5-HIAA secretion, and the respective ratio (5-HT/5-HIAA) in the ileum and medulla of rats. Control: saline, p. o.; Ond: 1.66 mg/kg, p. o.; Apr: 26.2 mg/kg p. o.; DAS: 2 mg/kg, p. o. Ond and Apr were administered 30 min before DAS. The ileum and medulla were collected 4 h after treatment (A and B- ileum; D and E - medulla). The concentrations of 5-HT and 5-HIAA were assessed using HPLC-ECD. The data are expressed as mean ± SEM. Statistical significance was assessed using the one-way ANOVA. ^*^ *p* < 0.05, ^**^ *p* < 0.01, ^***^ *p* < 0.001 vs. the control group, and ^#^ *p* < 0.05, ^# #^ *p* < 0.01, ^# # #^ *p* < 0.001 vs. the DAS group.

To confirm the involvement of the 5-HT and SP signaling pathways in DAS-induced emesis, we investigated the effect of the 5-HT_3_ receptor antagonist Ond and the NK_1_-R antagonist Apr on neurotransmitter and related receptor levels in the ileum and medulla of rat after DAS treatment. As shown in Figure 7, DAS significantly increased the release of 5-HT in the ileum and medulla oblongata (vs. control, *p* < 0.001), but had no significant effect on 5-HIAA content. The 5-HT_3_ receptor antagonist Ond significantly reduced the release of 5-HT in the ileum and medulla oblongata (vs. DAS, *p* < 0.01, *p* < 0.001), and significantly increased the 5-HIAA content in the ileum (vs DAS, *p* < 0.05). No significant effect on 5-HIAA content was observed in the medulla oblongata (vs DAS, *p* > 0.05). Of note, although Ond had no significant effect on 5-HIAA in the medulla oblongata, the 5-HIAA/5-HT ratio, which reflects the metabolic rate of 5-HT, was significantly reversed in the ileum and the medulla oblongata (vs. DAS, *p* < 0.01, *p* < 0.001, respectively), due to the relatively low medulla oblongata 5-HT levels.

Interestingly, the effect of the NK_1_ receptor antagonist Apr on DAS-induced 5-HT and 5-HIAA in the ileum and medulla oblongata was similar to that caused by Ond. However, the degree of Apr antagonism on 5-HT release in the medulla oblongata was lower than that of Ond. Further, the 5-HIAA/5-HT ratio did not significantly reverse in the medulla oblongata (vs DAS, *p* > 0.05).

We further investigated the effect of combined Ond and Apr treatment on 5-HT synthesis and metabolism. The drug combination could synergistically reduce the release of 5-HT in the ileum and medulla oblongata (vs. DAS, *p* < 0.001), and increase the 5-HIAA content in the ileum (vs. DAS, *p* < 0.001). However, there was no significant effect on 5-HIAA in the medulla oblongata (vs. DAS, *p* > 0.05). Of note, the 5-HIAA/5-HT ratio was significantly reversed in both tissues (vs. DAS, *p* < 0.001), suggesting that the combination of Ond and Apr can synergistically improve the DAS-induced increase of 5-HT.

### Effect of 5-HT_3_ or NK_1_ Receptor Antagonists on the DAS-Induced Alteration in *PPT-A* mRNA and SP Protein Levels in the Ileum and Medulla of Rats.

**FIGURE 8:**
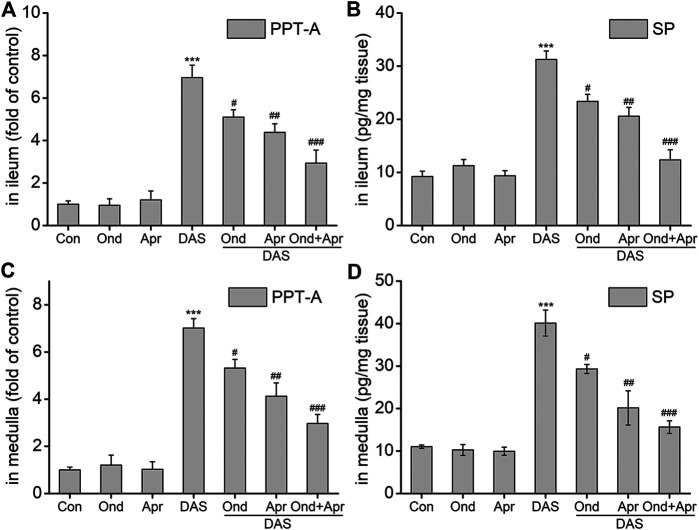
The effect of Ond and Apr on the DAS-induced PPT-A mRNA and SP protein levels in the ileum and medulla of rats. Control: saline, p. o.; Ond: 1.66 mg/kg, p. o.; Apr: 26.2 mg/kg p. o.; DAS: 2 mg/kg, p. o. Ond and Apr were administered 30 min before DAS. The ileum and medulla were collected 4 h after treatment (A and B- ileum; D and E - medulla). *PPT-A* mRNA levels were detected using quantitative real-time PCR. S*p* concentration was detected using a commercial assay kit. The data are expressed as mean ± SEM. Statistical significance was assessed using the one-way ANOVA. ^*^ *p* < 0.05, ^**^ *p* < 0.01, ^***^ *p* < 0.001 vs. the control group, and ^#^ *p* < 0.05, ^# #^ *p* < 0.01, ^# # #^ *p* < 0.001 vs. the DAS group.

To examine whether the DAS-induced emesis is associated with SP expression in the ileum and medulla, SP protein, and *PPT-A* mRNA levels were evaluated using ELISA and RT-PCR, respectively. As shown in Figures 8A–D, SP protein expression and *PPT-A* mRNA expression in the ileum and medulla oblongata were significantly increased after DAS treatment (vs. control, *p* < 0.001). Moreover, treatment with Ond or Apr significantly reduced their expression in the two tissues, but the effect of the NK_1_ receptor antagonist Apr was slightly stronger than that of Ond (vs. DAS, *p* < 0.01, *p* < 0.05, respectively). Of note, the combination of Ond and Apr further reduced SP protein and *PPT-A* mRNA expression in the two tissues (vs. DAS, *p* < 0.001).

### Effect of 5-HT_3_ and NK_1_ Receptor Antagonists on the DAS-Induced the Expression of 5-HT_3_ and NK_1_ Receptors in the Ileum and Medulla of Rats.

**FIGURE 9:**
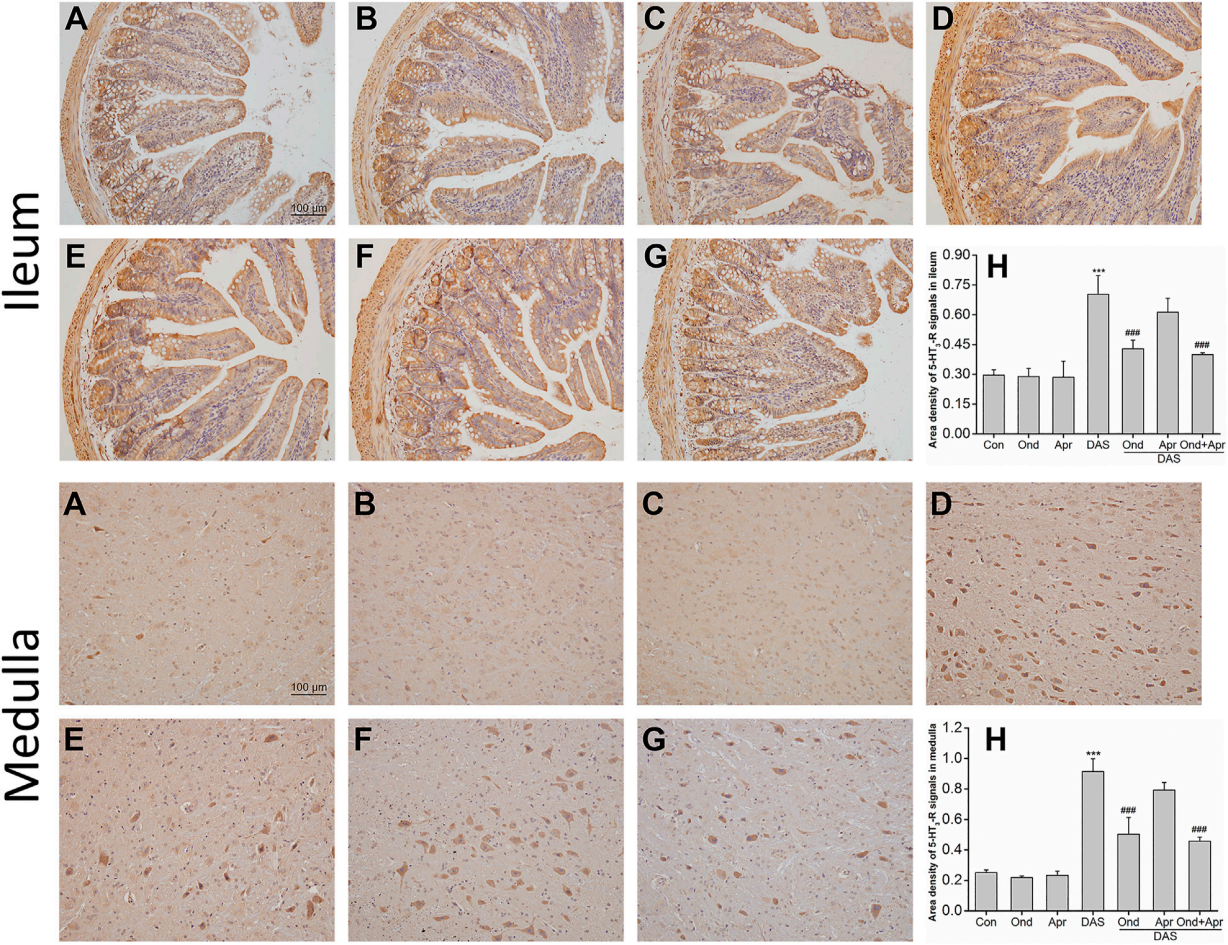
**T**he effect of Ond and Apr on DAS-induced 5-HT_3_ receptor expression in the ileum and medulla of rats, 4 h after treatment: immunohistochemical (IHC) analysis **(A)** Control **(B)** Ond 1.66 mg/kg **(C)** Apr 26.2 mg/kg **(D)** DAS 2 mg/kg **(E)** DAS 2 mg/kg + Ond 1.66 mg/kg **(F)** DAS 2 mg/kg + Apr 26.2 mg/kg **(G)** DAS 2 mg/kg + Ond 1.66 mg/kg + Apr 26.2 mg/kg. Ond and Apr were administered 30 min before DAS. Semi-quantitative analysis of the ratio of 5-HT_3_ receptor positive staining to the total field **(H)**. Data are expressed as mean ± SEM. Statistical significance was assessed using the one-way ANOVA. ^*^ *p* < 0.05, ^**^ *p* < 0.01, ^***^ *p* < 0.001 vs. the control group, and ^#^ *p* < 0.05, ^# #^ *p* < 0.01, ^# # #^ *p* < 0.001 vs. the DAS group.

The effect of Ond and Apr on the DAS-induced expression of 5-HT_3_ and NK_1_ receptors in the ileum and medulla of rats was observed using Immunohistochemistry (IHC). As shown in Figure 9, the expression of 5-HT_3_ receptors in the ileum mucosa and submucosa in the DAS-treated group was significantly higher than that in the NC group (*p* < 0.001, Figures 9A,D). Of note, the 5-HT_3_ receptor upregulation was confirmed by semi-quantitative analysis (Figure 9H). On the other hand, Ond or Apr treated groups (single treatments) did not show any changes in the expression of 5-HT_3_ receptor compared to the NC group (*p* > 0.05, Figures 9B,C). However, the 5-HT_3_ receptor expression levels in the DAS groups treated with Ond or the combination of Ond and Apr were significantly lower than those in the DAS group (*p* < 0.001, *p* < 0.001, Figures 9E,G,H). However, the NK_1_ receptor antagonist Apr alone did not reduce the 5-HT_3_ receptor expression induced by DAS (*p* > 0.05, Figures 9F,H).

The effects of Ond or Apr on the DAS-induced 5-HT_3_ receptor expression in the medulla were similar to those observed in the ileum. As shown in Figure 9 (medulla), the 5-HT_3_ receptor expression in neurons in the DAS group was significantly higher than that in the NC group (*p* < 0.001, Figures 9A,D). Again, the 5-HT_3_ receptor upregulation was confirmed by semi-quantitative analysis (Figure 9H). The expression of the 5-HT_3_ receptor in the Ond or Apr group was also not significantly different than that in the control group (*p* > 0.05, Figures 9B,C). Ond or the combination of Ond and Apr markedly suppressed the expression of 5-HT_3_ receptor in neurons from the medulla compared to that in the DAS group (*p* < 0.001, *p* < 0.001, Figures 9E,G,H). However (and once more), Apr alone did not reduce 5-HT_3_ receptor expression induced by DAS (*p* > 0.05, Figures 9F,H).

**FIGURE 10:**
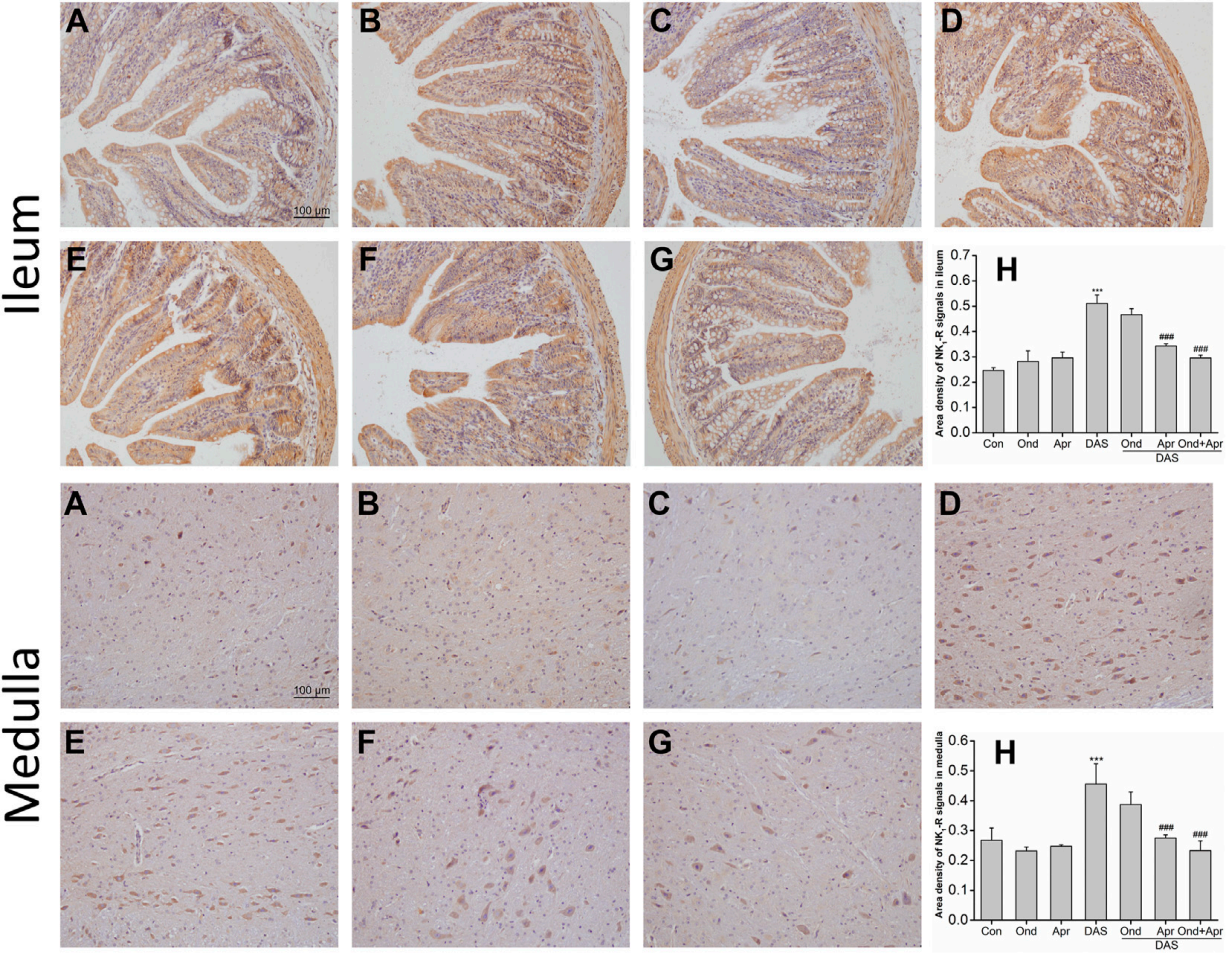
The effect of Ond and Apr on DAS-induced NK_1_ receptor expression in the ileum and medulla of rats, 4 h after treatment: IHC analysis **(A)** Control **(B)** Ond 1.66 mg/kg **(C)** Apr 26.2 mg/kg **(D)** DAS 2 mg/kg **(E)** DAS 2 mg/kg + Ond 1.66 mg/kg **(F)** DAS 2 mg/kg + Apr 26.2 mg/kg **(G)** DAS 2 mg/kg + Ond 1.66 mg/kg + Apr 26.2 mg/kg. Ond and Apr were administered 30 min before DAS. Semi-quantitative analysis of the ratio of NK_1_R positive staining to the total field **(H)**. Data are expressed as mean ± SEM. Statistical significance was assessed using the one-way ANOVA. ^*^ *p* < 0.05, ^**^ *p* < 0.01, ^***^ *p* < 0.001 vs. the control group, and ^#^ *p* < 0.05, ^# #^ *p* < 0.01, ^# # #^ *p* < 0.001 vs. the DAS group.

As shown in Figure 10 (ileum), IHC staining demonstrated that the expression of NK_1_ receptor in the mucosa and submucosa of the ileum in the DAS-alone group was significantly higher than that in the control group (*p* < 0.001, Figure 10A). The upregulation of the NK_1_ receptor was confirmed by semi-quantitative analysis (Figure 10H). Ond or Apr alone did not change the expression of NK_1_ receptor compared to that in the NC group (*p* > 0.05, Figure 10B). However, Apr alone, or in combination with Ond significantly suppressed the NK_1_ receptor expression induced by DAS (*p* < 0.001, *p* < 0.001, Figure 10F). Of note, the 5-HT_3_ receptor antagonist Ond alone had no effect on the NK_1_-R expression induced by DAS (*p* > 0.05, Figure 10E). Additionally, no obvious synergism was observed between Ond and Apr.

The effects of Ond or Apr on DAS-induced NK_1_ receptor expression in the medulla were similar to those observed in the ileum. As shown in Figure 10 (medulla), IHC staining demonstrated that NK_1_ receptor expression in neurons of the medulla of rats in the DAS group was significantly higher than that in the NC group (*p* < 0.001, Figure 10A). NK_1_ receptor upregulation was confirmed by semi-quantitative analysis (Figure 10H). Once more, the expression of the NK_1_ receptor in the Ond or Apr single-treated groups was not significantly different than that in the control group (*p* > 0.05, Figure 10B). However, Apr or the combination of Ond and Apr markedly suppressed the expression of NK_1_ receptor in neurons of the medulla compared to that in the DAS group (*p* < 0.001, *p* < 0.001, Figure 10F). On the other hand, Ond alone did not change the expression of the NK_1_ receptor induced by DAS (*p* > 0.05, Figure 10E). Further, no obvious synergism was observed between Ond and Apr.

## Discussion

### Establishment of a Rat Pica Model Using DDP

Changshan is a good anti-malarial medicine according to traditional Chinese medicine, but it has not been used as a drug due to its severe vomiting side effects. Although previous studies used pigeons, dogs, and other models to explore dichroine-related vomiting, the underlying mechanism of dichroine-induced emesis has not yet been elucidated. Jiang’s research on the emetic mechanism of dichroine using pigeon and dog models (Chiang et al., 1957; Chiang, 1961) did not reach the same conclusion as that in research performed by K. K. Chen, using pigeons (Henderson and Rose, 1949). Since dogs quickly learn that a medicine causes vomiting, the vomiting phenomenon may occur even if no more vomiting agent is given. Additionally, pigeons, unlike cats and dogs, lack a chemosensitivity zone; therefore, it is difficult to judge whether vomiting occurs in pigeons via direct action on the central nervous system. In addition, because of limited sample-sizes, sufficient experimental data have not been obtained in these studies. Thus, it remains controversial whether DAS-induced vomiting occurs via stimulation of the peripheral nervous system or via direct action on the central nervous system. Therefore, to warrant a sufficient sample-size and reproducibility, the present study explored the mechanism involved in DAS-induced emesis using a surrogate rat pica model.

Rodents, such as rats and mice, are unable to vomit, since they have no vomiting reflex. However, Mitchell et al. (Mitchell et al., 1976; Mitchell et al., 1977) found that several emetic agents elicit dose-dependent pica in rats, while anti-emetic agents dose-dependently inhibit the occurrence of pica behavior. Takeda et al. further defined the concept of pica as an eating disorder in rats that may be analogous to gastrointestinal discomfort such as nausea and vomiting (Ossenkopp, 1983; Takeda et al., 1993). Later, many scholars (Yamamoto et al., 2011; Takano et al., 2014; Wojnicz et al., 2016) also clearly pointed out that rat eating disorders are equivalent to nausea and vomiting behaviors of other animals with vomiting reflexes (to some extent). Importantly, pica in rats is believed to be mediated by the same mechanisms (and the same receptors) responsible for vomiting in humans and other species (Takeda et al., 1993). Studies have further shown that rats have similar neurotransmitter changes in brain regions as emetic animals when stimulated by emetic agents (Takeda et al., 1995). Therefore, the kaolin ingestion model may offer a simplistic approach to characterize drug-induced vomiting and studying the underlying vomiting mechanisms. Previous studies reported that the incidence, latency, and duration of pica behavior in rats induced by the chemotherapeutic drug DDP are consistent with clinical vomiting symptoms and can reproduce the clinical therapeutic effects of antiemetic drugs (Takano et al., 2014). Further, the involvement of SP in the development of DDP-induced acute and delayed emesis was studied using the pica rat model (Yamamoto et al., 2002), and satisfactory results were obtained. Thus, the pica model can qualitatively and quantitatively characterize the degree of vomiting induced by an emetic agent and allows the in-depth study of the vomiting mechanism. Considering that DAS is as effective as DDP with a maximum emetic potential (Mehendale et al., 2004; Wang et al., 2005), DAS may also potentially induce pica in rats. Therefore, we used a rat pica model to characterize DAS-induced vomiting and to investigate the possible underlying mechanism.

First, the kaolin formulation and preparation were investigated using DDP. The kaolin preparation is a key issue that determines the success or failure of the pica model: the kaolin texture can neither be too hard nor too soft. If the kaolin is too soft, powdery debris appear when rats gnaw on the kaolin, leading to large weight changes and false-positive results. On the other hand, if the kaolin is too hard, the rats will bite hard (especially when the drug is toxic and the animal is in poor condition) and kaolin intake will decrease abnormally, resulting in false negatives. Therefore, based on preliminary experimental results and literature reports, this study optimized the kaolin preparation method and prepared moderately soft and hard kaolin, which produced almost no detritus during the biting process and stimulated the rats to eat the most kaolin with the same drug dose. In addition, general rat health is an important factor affecting kaolin intake. When the dose is too high, drug toxicity may cause systemic damage, leading to poor animal condition, and both kaolin and normal food intake is significantly reduced. In this context, kaolin intake cannot objectively and effectively reflect the degree of vomiting in rats. On the other hand, if the drug dose is too low, drug-induced kaolin intake may be limited (similar to the usual in the context of rat exploration), resulting in no obvious statistically significant differences. Therefore, in pica experiments, kaolin preparations should be strictly controlled to ensure an optimal texture. Moreover, the appropriate drug dose should also be defined to ensure that the animal can maintain a good general state, retaining, however, a clear obvious pica behavior, so that the kaolin ingestion can objectively and accurately reflect the drug dose.

The modified kaolin feed was used to observe rat pica induced by different DDP doses. The rats in the NC group showed an obvious pica behavior on the first day of the adaptation period. However, kaolin intake decreased sharply on the second adaptation day and was almost zero on the third day (data not shown). This phenomenon may be due to the natural curiosity of rats, leading to kaolin intake when the rats are first given kaolin. When the rats adapted to the presence of kaolin, kaolin intake decreased significantly. Hence, we followed a three- or 5 days adaptation period prior to the study period to ensure that the rats had no obvious pica behavior before the formal experiment. DDP significantly increased the rats’ pica behavior, and kaolin intake was inversely related to normal food intake; on the other hand, the rats’ body weight was positively correlated with the normal food intake. Thus, rats are sensitive to DDP and can effectively be used characterize (in a surrogate fashion) drug-induced vomiting. Because kaolin intake in rats treated with 6 mg/kg DDP was more obvious, lasted for the entire observation period, and the animals did not die during the experiment, 6 mg/kg was used as the DDP positive-control dose in subsequent experiments.

### Analysis of the DAS-Induced Pica Behavior in Rats

Although pica models were used to investigate the vomiting mechanisms of emetic agents and in the development of antiemetic drugs, no study investigated the characteristics and mechanism of DAS-induced emesis using a rat pica model. Therefore, we first used saline as a negative control and 6 mg/kg DDP as a positive control to investigate whether DAS could induce pica in rats. The animals in the NC group showed no obvious kaolin intake during the experimental period, indicating that they have adapted to kaolin and did not consume it due to curiosity. On the other hand, the kaolin intake in the positive control group was significantly increased, suggesting that the rat pica model was successful. However, the kaolin intake induced by DDP in this experiment decreased significantly on the first day after treatment. Further, diarrhea in the DDP group was more serious than that observed in the first experiment, possibly because different batches of animals may have different status, thus affecting kaolin intake. This should be noted in future experiments; this said, these differences did not affect any conclusions reached in this study.

Remarkably, administration of 2, 4, and 6 mg/kg of DAS caused pica behavior in rats, indicated by a significant increase in kaolin consumption post-treatment. Interestingly, there was a difference in the development of DAS-induced pica between doses of 2 and 4 mg/kg; 2 mg/kg DAS induced a biphasic kaolin intake profile, whereas higher DAS doses (4 and 6 mg/kg) showed a single peak profile, indicating that the mechanism of different doses of DAS-induced pica behavior may differ. This is expected, as we observed similar situations in other cytotoxic drugs such as DDP. Acute phase emesis occurs within 24 h after drug administration, whereas delayed emesis begins 24 h after drug administration. The dose of 5 mg/kg DDP can induce obvious acute-phase vomiting and delayed-phase vomiting in ferrets, but the higher dose of 10 mg/kg DDP can only induce acute phase vomiting because it causes toxic effects (Rudd et al., 1994). There are also reports showing that the greatest amount of pica/kaolin consumption was induced by lower rather than by higher DDP doses (Takeda et al., 1995; Aung et al., 2003). Although this effect of DDP has not been systematically studied, observation of the animals’ general state and pathological examinations suggested the generalized effects of the cytotoxic treatment (Wynn et al., 1993; Rudd et al., 1994; Rudd et al., 2006; Zhu et al., 2009; Percie du Sert et al., 2011; Yamamoto et al., 2014). Additionally, DAS is a toxic substance that causes nausea, vomiting, diarrhea, gastrointestinal tract pathological injuries, and hydropic degeneration of the liver in animals in the nanomolar range; larger doses may even affect other systems (Henderson and Rose, 1949). Our study of dose-time-toxicity induced by DAS based on mouse models showed (data not published) that with increasing doses, in the gastrointestinal tract, DAS did not simply cause irritation, but also caused the development of more serious intestinal ulcers and bleeding. When the dose of DAS exceeds a certain critical value, other organs may become involved, such as atrophy of the spleen and thymus gland in animals, suggesting that with dose increases, DAS can damage the digestive system as well as other systems and even cause death. In this study, animals treated with different DAS doses showed different degrees of diarrhea that worsened in a dose-dependent manner; this was also true for food intake and body weight, decreasing in a dose-dependent manner. With higher doses of DAS, we observed diarrhea, lethargy, and less dynamic movements, suggesting that there may be functional or even organic lesions in the internal organs of the animals. Thus, the toxic side effects may not be simply characterized by vomiting. These results suggest that when the dose of DAS is equal to or higher than 4 mg/kg, the drug toxicity is substantial, causing damage to multiple organs and affecting normal feeding behavior, leading to an abnormal inverse correlation with kaolin intake, and the specific mechanism remains to be investigated. It is necessary to pay special attention to the drug dosage, when using the pica model to characterize vomiting. Therefore, considering the feeding behavior and body weight of rats, the lower dose of DAS at 2 mg/kg objectively represented both acute and delayed pica behavior. Based on these results, this dose was selected to evaluate the effects of antiemetic treatments on DAS-induced pica.

### The Involvement of 5-HT- and SP-Related Pathways in DAS-Induced Vomiting.

It is common to explore the mechanism of action of unknown compounds testing their compatibility with drugs whose mechanism of action is clear. Yamamoto et al. (Yamamoto et al., 2002) explored the role of SP in DDP-induced acute and delayed vomiting by combining 5-HT_3_ and NK_1_ receptor antagonists and found that SP produced in the medulla oblongata plays a role in DDP-induced pica. Moreover, Chiang (Chiang et al., 1957) analyzed the vomiting mechanism of dichroine, observing the compatibility of dichroine and chlorpromazine, and using the effects of chlorpromazine on digitalis glycoside and copper sulfate-induced vomiting in pigeons as a control. This study indicated that the emetic mechanism of dichroine is two-fold: a peripheral reflex and a direct effect on the vomiting center. Investigations over the past 3 decades gradually elucidated the clinical significance of several neurotransmitters in the emetic process. Even though multiple neurotransmitters are involved in triggering emesis, dopamine, 5-HT, and substance *p* play the largest roles. Their receptors are mainly the DA receptor, NK_1_ receptor, and 5-HT_3_ receptor, respectively, which stimulate the vagus nerve and finally act on the vomiting center, causing vomiting. This fact has become the new hotspot for research on vomiting mechanisms (Rojas and Slusher, 2012). Therefore, this study draws on the previous research ideas of compatibility and uses new achievements of modern vomiting research to explore the possible mechanism of vomiting induced by DAS by combining DAS with three antiemetic agents with a clear mechanism of action.

First, the rat pica model was used to observe changes in kaolin intake before and after the combination of DAS with different antiemetic agents and consequently to explore the possible vomiting pathways of DAS. The 5-HT_3_ receptor antagonist Ond and the NK_1_ receptor antagonist Apr significantly reduced the increase of kaolin intake induced by DAS. The response of Ond had a significant dose-dependent relationship, while Apr showed no significant dose-dependency. The DA antagonist Met had no significant effect on DAS-induced pica behavior. These results suggest that 5-HT_3_ receptor- and NK_1_ receptor-induced vomiting pathways may play an important role in DAS-induced vomiting. Further observation of the effect of the antiemetics on normal food intake induced by DAS showed that Ond and Apr effectively antagonized DAS-induced emesis and significantly improved DAS-induced anorexia. Therefore, in future experiments, the key links between these two pathways will be tested to verify whether DAS can indeed cause emetic effects through these two pathways.

#### The Role of 5-HT-induced Network Pathway in DAS-Induced Vomiting

One of the most important advances in research on chemotherapy-induced nausea and vomiting was the disclosure of the critical role played by 5-HT. 5-HT is a monoamine neurotransmitter present in the central and peripheral nervous systems. Of the multiple 5-HT receptors identified to date, the 5-HT_3_ receptor appears to be the most important in the acute phase of chemotherapy-induced nausea and vomiting (Darmani et al., 2009). Recent studies showed that 5-HT released from the gastrointestinal (GI) tract and brain acts on vagal afferents containing 5-HT_3_ receptors (Darmani and Ray, 2009). These activate specific nuclei in the medulla (Bhandari et al., 1992; Andrews and Horn, 2006) to induce emesis. Selective antagonists of the 5-HT_3_ receptor are currently the most effective class of antiemetics for the prevention of acute chemotherapy-induced nausea and vomiting (Higgins et al., 1989; Miller and Nonaka, 1992; Jordan et al., 2007). Therefore, to clarify the role of the 5-HT_3_ receptor pathway in DAS-induced vomiting, changes in 5-HT and 5-HT_3_ receptor after a single dose of DAS in the context of compatibility with known antiemetics were compared. 5-HT and *5-HT*_*3*_ receptor gene expression after DAS administration were significantly increased in the peripheral ileum and central medulla oblongata at 2, 4, 8, and 24 h, while the 5-HT metabolite 5-HIAA was not significantly changed in the two tissues. This suggests that DAS increases the 5-HT content by promoting 5-HT secretion and inducing vomiting. At the same time, via the increase of the *5-HT*_*3*_ receptor expression, the intensity of 5-HT-induced vomiting was enhanced.

In the pica experiment, we observed that the pica behavior in rats induced by DAS mainly occurred at approximately 4 h, and diarrhea generally began 4 h after treatment. Therefore, in the subsequent compatibility experiments, the sampling time point selected was precisely 4 h after DAS administration. In addition, the time-effect relationship study also found that the changes in 5-HT and 5-HT_3_ receptor levels induced by DAS mainly occurred in the acute phase and reached a peak, four or 8 h after drug administration. Of note, there was no significant effect on their expression levels at 48 and 72 h post-treatment. Similarly, the *5-HT*_*3*_ receptor gene expression in the medulla oblongata peaked 8 h after drug administration, and the expression peaks of other indicators in both the ileum and the medulla oblongata appeared at 4 h post-treatment. The 5-HT pathway may play an important role in the rat acute-phase pica behavior induced by DAS.

Further, observing the effects of Ond and Apr, individually, and their combination on DAS-induced neurotransmitters and their receptors, we proved that indeed DAS induces pica behavior in rats. We found that Ond effectively reversed the DAS-induced increase in 5-HT in the ileum, while also significantly increasing the content of 5-HTAA. However, no significant effect was observed with respect to 5-HIAA in the medulla oblongata. We also observed a significant reversal in the 5-HIAA/5-HT ratio in the two tissues. Overall, these results suggest that Ond can improve the DAS-induced pica behavior in rats via the prevention of 5-HT upregulation in the peripheral and central centers. Apr also significantly reduced the DAS-induced content of 5-HT in the ileum and medulla oblongata, and reversed the 5-HIAA/5-HT ratio; however, the degree was lower than that observed with Ond. Furthermore, in the medulla oblongata, Apr only reduced the absolute 5-HT content without significantly influencing 5-HIAA, and could not significantly reverse the 5-HIAA/5-HT ratio. These results suggest that the influence of Apr on 5-HT synthesis in the ileum was greater than that in the medulla oblongata. Further, the combined treatment of Ond and Apr could synergistically reduce the DAS-induced 5-HT increase in the ileum and medulla oblongata and significantly reverse the 5-HIAA/5-HT ratio. Of note, the degree of reversal was far higher than that caused by Ond alone. However, there was no significant effect on 5-HIAA content in the medulla oblongata. This phenomenon may be because approximately 95% of the intestinal 5-HT content is present in enterochromaffin cells in the intestinal mucosa (Hirafuji et al., 2000; Andrews and Horn, 2006) and the amount of 5-HT secreted in the brain is relatively low. The blood-brain barrier in the brain likely reduces the bioavailability of the drug (Daneman and Prat, 2015), thus making the effect of intestinal 5-HT in the brain weaker than in that in the ileum.

Immunohistochemistry was used to study the effects of antiemetic agents on 5-HT_3_ receptor protein expression. The results showed that DAS alone significantly increased the 5-HT_3_ receptor content in the ileum and medulla oblongata. Ond significantly reduced 5-HT_3_ expression in the two tissues, while Apr has no significant effect. Moreover, the combination of Ond and Apr had no clear additive effects. This suggests that 5-HT_3_ receptors play a key role in DAS-induced vomiting, but only the specific 5-HT_3_ receptor antagonist Ond can effectively inhibit their expression. In summary, the 5-HT_3_ receptor-induced vomiting network plays an important role in DAS-induced acute-phase vomiting, mainly due to significantly increased 5-HT and 5-HT_3_ receptor content in the peripheral ileum and central medulla.

#### The Role of SP-Induced Network Pathway in DAS-Induced Vomiting

SP is an 11 amino acid peptide synthesized from PPT-A protein precursors, and it is widely distributed in the CNS and peripheral nervous systems (Ribeiro-da-Silva and Hökfelt, 2000). In the peripheral nervous system, SP is found in the terminals of primary sensory neurons and neurons intrinsic to the gastrointestinal (GI) tract. SP increases vagal afferent activity via adjacent NK_1_ receptors (Zhong et al., 2016). Furthermore, SP co-localizes with 5-HT within the enterochromaffin cells of the gastrointestinal tract. These reports strongly suggest that SP is involved during several stages of the emetic pathway. Therefore, we determined the role of SP and NK_1_ receptors in DAS-induced vomiting. Our results showed that the changes in SP and NK_1_ receptor showed similar characteristics as the 5-HT_3_ pathway. Changes in SP, its precursor protein PPT-A, and *NK*_*1*_ receptor mRNA expression significantly increased within 24 h of drug treatment in the ileum and medulla oblongata. In addition, *NK*_*1*_ receptor mRNA expression in the medulla oblongata peaked 8 h after drug-treatment, while all other indicators peaked 4 h after treatment. Therefore, in the subsequent compatibility experiments, we selected the 4 h time-point as the most appropriate one.

Further, observations of the expression of key vomiting neurotransmitters and receptors in the NK_1_ receptor pathway induced by DAS, in the context of Ond and Apr single treatments or of their combination showed that Ond significantly reduced the DAS-induced *SP* and *PPT-A* mRNA upregulation in the ileum and medulla oblongata. However, there was no significant effect on the DAS-induced NK_1_ receptor expression in the two tissues. This suggests that the 5-HT_3_ receptor-specific antagonist Ond only affected SP expression, and had no significant effect on the expression of the NK_1_ receptor. Importantly, Apr significantly reduced SP protein and PPT-A mRNA expression induced by DAS, and significantly reduced the DAS-induced NK_1_ receptor expression in the two tissues. The combination of Ond and Apr could further synergistically reduce SP and PPT-A in the two tissues, but the effect on the NK_1_ receptor expression was equivalent to that of Apr, indicating no synergistic effect. In summary, the NK_1_ receptor-induced vomiting network plays an important role in DAS-induced acute-phase pica behavior, mainly manifested by a significant increase in the expression of SP protein and NK_1_ receptors in the peripheral ileum and central medulla oblongata. Previous reports suggest an interaction between the 5-HT_3_ and NK_1_ receptor pathways (Rojas and Slusher, 2012). This potential interaction allows the vomiting effects of the two pathways to exert a cascade amplification effect which makes DAS induce more severe vomiting than that expected.

#### The Interaction Between 5-HT_3_ and NK_1_ Receptor Pathways

Through the systematic study of the 5-HT and SP pathways in DAS-induced vomiting, we found that Ond and Apr had obvious cross-antagonistic effects on the elevation of neurotransmitters (5-HT and SP) induced by DAS, but no significant cross-antagonism on the two respective receptors. This suggests the coexistence of 5-HT and SP in the same cell, and further suggests that the two neurotransmitters are released simultaneously when the drug stimulates the corresponding cells (Rubenstein et al., 2006). However, the synergistic changes in the two receptors are not necessarily triggered. A study on the second-generation 5-HT_3_ receptor-specific antagonist palonosetron, which simultaneously antagonizes acute and delayed vomiting, found that palonosetron antagonized acute vomiting mainly by binding to the 5-HT_3_ receptor on the cell surface. While palonosetron binds to the 5-HT_3_ receptor, it also induces the internalization of the NK_1_ receptor, thus playing an antagonistic role against delayed vomiting (Rojas et al., 2010). Therefore, it is understandable that Ond and Apr in this study showed cross-inhibitory effects on the two vomiting neurotransmitters (5-HT and SP), but had no significant cross-influence on the respective receptors.

Although we demonstrated the role of 5-HT and SP neurotransmitter pathways in DAS-induced rat acute-phase pica behavior, tour results do not explain the mechanism of DAS-induced delayed-phase pica behavior. Notably, when investigating the mechanism of delayed-phase pica behavior, although we selected pica behavior which was more noticeable 36, 40, 48, 60, 72, 80, and 96 h, etc., no significant changes were observed in 5-HT and SP neurotransmitter. Therefore, our results only show the data at two representative time points of 48 and 72h, suggesting that the mechanism of delayed-phase pica behavior induced by DAS differs from that of the acute-phase. This is also demonstrated by the completely different acute and delayed pica behavior induced by 2mg/kg DAS. This phenomenon has also been observed in more advanced ferret vomiting models. In the ferret model, the degree and frequency of acute-phase vomiting induced by DAS is significantly stronger than those of delayed-phase vomiting, and the acute-phase animal vomit is ejected, and vomiting sounds were clearly heard. However, the number of vomiting events in the delayed phase is greatly reduced and only a small amount of fluid was vomited. This suggests that the mechanism of acute- and delayed-phase vomiting induced by DAS may not be the same. Therefore, these behaviors must be explored from other perspectives. This may also explain why we did not directly detect changes in 5-HT and SP pathway-related neurotransmitters and receptors during the delay period. A similar situation was reported for the cytotoxic drug DDP. In 2014, Yamamoto used a rat pica model and newly developed *in vivo* brain microdialysis approach to dynamically collect the nucleus of the solitary tract (NTS) of rats in real-time to examine changes in SP release and determined the SP production involved in the development of the delayed phase of DDP-induced pica (Yamamoto et al., 2014). In this experiment, we only detected the content of 5-HT and SP in the medulla oblongata and ileum tissues, and did not detect the real-time changes in related neurotransmitters in the NTS. Therefore, it is unclear whether the 5-HT and SP pathways play a role in delayed phase pica induced by DAS. In addition, receptor activity is closely related to the expression location of these receptors in cells. For example, internalization of receptors can cause a reduction in the receptor population at the cell surface and result in persistent inhibition of receptor function. The second-generation 5-HT_3_ receptor antagonist Palonosetron (Pal) acts through this mode of action to antagonize the delayed vomiting effect induced by DDP. To determine whether the mechanism of delayed-phase vomiting induced by DAS is similar, we will perform immunofluorescence in our subsequent experiments to further clarify the effect of DAS on the expression and location of 5-HT_3_ and NK_1_ receptors on the cell surface, combined with neurotransmitters in NTS qualitative dynamic detection. We will also examine the mechanism of delayed vomiting induced by DAS. In summary, our results suggest that the underlying mechanism of DAS-induced delayed phase behavior occurs because of the secondary effects of DAS-induced receptor externalization and the increase in SP release in the NTS; however, further experimental verification is needed.

The above results show that the rat pica model can be used to characterize DAS-induced vomiting and the underlying mechanism. The 5-HT and SP-pathways’ mediated DAS-induced acute emesis in the pica rat model play an important role. A novel result of this study is the finding that the 5-HT_3_ and NK_1_ receptor-specific antagonists Ond and Apr can effectively antagonize DAS-induced acute pica behavior. This suggests a potential therapeutic strategy for the treatment of DAS-induced acute emesis reactions. Finally, pica in rats can be used as a good alternative rodent model to dogs, cats, or pigeons, particularly for preliminary and rapid screening of antiemetic agents.

## Data Availability

The raw data supporting the conclusion of this article will be made available by the authors, without undue reservation, to any qualified researcher.

## References

[B1] Andrews P. L. R. Horn C. C. (2006). Signals for nausea and emesis: implications for models of upper gastrointestinal diseases. Auton. Neurosci. 125, 100–115. 10.1016/j.autneu.2006.01.008 16556512 PMC2658708

[B2] Ashley E. A. Dhorda M. Fairhurst R. M. Amaratunga C. Lim P. Suon S. (2014). Spread of artemisinin resistance in Plasmodium falciparum malaria. N. Engl. J. Med. 371, 411–423. 10.1056/NEJMoa1314981 25075834 PMC4143591

[B3] Aung H. H. Dey L. Mehendale S. Xie J. T. Wu J. A. Yuan C. S. (2003). Scutellaria baicalensis extract decreases cisplatin-induced pica in rats. Cancer Chemother. Pharmacol., 52, 453–458. 10.1007/s00280-003-0694-9 12942313

[B4] Bhandari P. Bingham S. Andrews P. L. R. (1992). The neuropharmacology of loperamide-induced emesis in the ferret: the role of the area postrema, vagus, opiate and 5-HT3 receptors. Neuropharmacology 31, 735–742. 10.1016/0028-3908(92)90034-m 1326727

[B5] Chiang W. T. instructor Jang C. S. Yang Z. C. (1957). Studies of the emetic action of Alkaloids of Ch’ang Shan alkaloid. I. The mechanism of pigeon-emesis by alkaloids of Ch’angshan. Acta Acad. Med. Primae Shanghai 3, 253–257. CNKI:SUN:SHYK.0.1957-03-025

[B6] Chiang W. T. (1961). The mechanism of the emetic action of β-dichroine in dogs. Acta Physiol. Sin. 24, 180–186. CNKI:SUN:SLXU.0.1961-Z1-004

[B7] Chou T. Q. Fu F. Y. Kao Y. S. (1948). Antimalarial constituents of Chinese drug, ch’ang Shan, Dichroa febrifuga Lour. J. Am. Chem. Soc. 70, 1765–1767. 10.1021/ja01185a028 18861766

[B8] Collaboration Research Group for Qinghaosu. (1977). A novel kind of sesquiterpene lactone-artemisinin. Chin. Sci. Bull. 22, 142. CNKI:SUN:KXTB.0.1977-03-011

[B9] Cook J. A. Choudhuri R. Degraff W. Gamson J. Mitchell J. B. (2010). Halofuginone enhances the radiation sensitivity of human tumor cell lines. Cancer Lett. 289, 119–126. 10.1016/j.canlet.2009.08.009 19713035 PMC2835928

[B10] Daneman R. Prat A. (2015). The blood-brain barrier. Cold Spring Harb Perspect. Biol. 7, a020412. 10.1101/cshperspect.a020412 25561720 PMC4292164

[B11] Darmani N. A. Crim J. L. Janoyan J. J. Abad J. Ramirez J. (2009). A re-evaluation of the neurotransmitter basis of chemotherapy-induced immediate and delayed vomiting: evidence from the least shrew. Brain Res. 1248, 40–58. 10.1016/j.brainres.2008.10.063 19022231

[B12] Darmani N. A. Ray A. P. (2009). Evidence for a re-evaluation of the neurochemical and anatomical bases of chemotherapy-induced vomiting. Chem. Rev. 109, 3158–3199. 10.1021/cr900117p 19522506

[B13] Dondorp A. M. Nosten F. Yi P. Yi P. Das D. Phyo A. P. Tarning J. (2009). Artemisinin resistance in Plasmodium falciparum Malaria. N. Engl. J. Med. 361, 455–467. 10.1056/nejmoa0808859 19641202 PMC3495232

[B14] Fakhfouri G. Mousavizadeh K. Mehr S. E. Dehpour A. R. Zirak M. R. Ghia J. E. (2015). From chemotherapy-induced emesis to neuroprotection: therapeutic opportunities for 5-HT3 receptor antagonists. Mol. Neurobiol. 52, 1670–1679. 10.1007/s12035-014-8957-5 25377794

[B15] Figueiredo-Pontes L. L. Lima A. S. G. Santana-Lemos B. A. Lange A. P. A. Oliveira L. C. Garcia A. B. (2007). Halofuginone exerts antiproliferative and antiangiogenic actions on acute promyelocytic leukemia cells through modulation of the TGFβ pathway. Blood 110, 2850. 10.1182/blood.V110.11.2850.2850

[B16] Fortin S. M. Chartoff E. H. Roitman M. F. (2016). The aversive agent lithium chloride suppresses phasic dopamine release through central GLP-1 receptors. Neuropsychopharmacol 41, 906–915. 10.1038/npp.2015.220 PMC4707837 26211731

[B17] Gan T. J. (2005). Selective serotonin 5-HT 3 receptor antagonists for postoperative nausea and vomiting CNS Drugs 19, 225–238. 10.2165/00023210-200519030-00004 15740177

[B18] Henderson F. G. Rose C. L. (1949). gamma-Dichroine, the antimalarial alkaloid of ch’ang Shan. J. Pharmacol. Exp. Ther. 95, 191–200. 18111493

[B19] Higgins G. A. Kilpatrick G. J. Bunce K. T. Jones B. J. Tyers M. B. (1989). 5-HT3 receptor antagonists injected into the area postrema inhibit cisplatin-induced emesis in the ferret. Br. J. Pharmacol. 97, 247–255. 10.1111/j.1476-5381.1989.tb11948.x 2720310 PMC1854462

[B20] Hirafuji M. Minami M. Endo T. Ogawa T. Parvez S. H. (2000). Intracellular regulatory mechanisms of 5-ht release from enterochromaffin cells in intestinal mucosa. Biogenic Amines 16, 29–52.

[B21] Hu W. P. You X. H. Guan B. C. Ru L. Q. Chen J. G. Li Z. W. (2004). Substance P potentiates 5-HT3 receptor-mediated current in rat trigeminal ganglion neurons. Neurosci. Lett. 365, 147–152. 10.1016/j.neulet.2004.04.072 15245797

[B22] Jang C. S. Fu F. Y. Wang C. Y. Huang K. C. Lu G. Chou T. C. 1946). Ch’ang Shan, a Chinese antimalarial herb. Science, 103, 59. 10.1126/science.103.2663.59-b 17835430

[B23] Jang C. S. Zhou T. C. (1955). Research on domestic antimalarial medicinal materials (1) changshan (clinical experimental report). Shanghai J. Tradit. Chin. Med. 8, 29. 10.16305/j.1007-1334.1955.08.009

[B24] Jiang S. Zeng Q. Gettayacamin M. Tungtaeng A. Wannaying S. Lim A. (2005). Antimalarial activities and therapeutic properties of febrifugine analogs. Aac 49, 1169–1176. 10.1128/AAC.49.3.1169-1176.2005 PMC549280 15728920

[B25] Jordan K. Schmoll H. J. Aapro M. S. (2007). Comparative activity of antiemetic drugs. Crit. Rev. Oncol./Hematol. 61, 162–175. 10.1016/j.critrevonc.2006.08.003 17208005

[B26] Koepfli J. B. Mead J. F. Brockman J. A. Jr. (1947). An alkaloid with high antimalarial activity from dichroa Febrifuga1. J. Am. Chem. Soc. 69, 1837. 10.1021/ja01199a513 20251439

[B27] Mehendale S. R. Aung H. H. Yin J. J. Lin E. Fishbein A. Wang C. Z. (2004). Effects of antioxidant herbs on chemotherapy-induced nausea and vomiting in a rat-pica model. Am. J. Chin. Med. 32, 897–905. 10.1142/S0192415X04002508 15673195

[B28] Miller A. D. Nonaka S. (1992). Mechanisms of vomiting induced by serotonin-3 receptor agonists in the cat: effect of vagotomy, splanchnicectomy or area postrema lesion. J. Pharmacol. Exp. Ther. 260, 509–517. 1738101

[B29] Mitchell D. Krusemark M. L. Hafner E. (1977). *Pica*: a species relevant behavioral assay of motion sickness in the rat. Physiol. Behav. 18, 125–130. 10.1016/0031-9384(77)90103-2 561970

[B30] Mitchell D. Wells C. Hoch N. Lind K. Woods S. C. Mitchell L. K. (1976). Poison induced pica in rats. Physiol. Behav. 17, 691–697. 10.1016/0031-9384(76)90171-2 1034944

[B31] Ossenkopp K. P. (1983). Area postrema lesions in rats enhance the magnitude of body rotation-induced conditioned taste aversions. Behav. Neural Biol. 38, 82–96. 10.1016/s0163-1047(83)90414-4 6626102

[B32] Pandey R. K. Kumbhar B. V. Srivastava S. Malik R. Sundar S. Kunwar A. (2017). Febrifugine analogues as Leishmania donovani trypanothione reductase inhibitors: binding energy analysis assisted by molecular docking, ADMET and molecular dynamics simulation. J. Biomol. Struct. Dyn. 35, 141–158. 10.1080/07391102.2015.1135298 27043972

[B33] Percie du Sert N. Rudd J. A. Apfel C. C. Andrews P. L. R. (2011). Cisplatin-induced emesis: systematic review and meta-analysis of the ferret model and the effects of 5-HT3 receptor antagonists. Cancer Chemother. Pharmacol. 67, 667–686. 10.1007/s00280-010-1339-4 20509026 PMC3043247

[B34] Ribeiro-da-Silva A. Hökfelt T. (2000). Neuroanatomical localisation of Substance P in the CNS and sensory neurons. Neuropeptides 34, 256–271. 10.1054/npep.2000.0834 11049730

[B35] Rojas C. Slusher B. S. (2012). Pharmacological mechanisms of 5-HT3 and tachykinin NK1 receptor antagonism to prevent chemotherapy-induced nausea and vomiting. Eur. J. Pharmacol. 684, 1–7. 10.1016/j.ejphar.2012.01.046 22425650

[B36] Rojas C. Thomas A. G. Alt J. Stathis M. Zhang J. Rubenstein E. B. (2010). Palonosetron triggers 5-HT3 receptor internalization and causes prolonged inhibition of receptor function. Eur. J. Pharmacol. 626, 193–199. 10.1016/j.ejphar.2009.10.002 19836386

[B37] Rubenstein E. B. Slusher B. S. Rojas C. Navari R. M. (2006). New approaches to chemotherapy-induced nausea and vomiting. Cancer J *.* 12, 341–347. 10.1097/00130404-200609000-00003 17034670

[B38] Rudd J. A. Jordan C. C. Naylor R. J. (1994). Profiles of emetic action of cisplatin in the ferret: a potential model of acute and delayed emesis. Eur. J. Pharmacol. 262, R1–R2. 10.1016/0014-2999(94)90048-5 7813558

[B39] Rudd J. A. Ngan M. P. Wai M. K. King A. G. Witherington J. Andrews P. L. R. (2006). Anti-emetic activity of ghrelin in ferrets exposed to the cytotoxic anti-cancer agent cisplatin. Neurosci. Lett. 392, 79–83. 10.1016/j.neulet.2005.08.062 16182445

[B140] Saito R. Takano Y. Kamiya H. O. (2003). Roles of substance P and NK1 receptor in the brainstem in the development of emesis. J. Pharmacol. Sci. 91, 87–94. 10.1254/jphs.91.87 12686752

[B40] Schank J. R. Heilig M. (2017). Substance P and the neurokinin-1 receptor: the new CRF. Int. Rev. Neurobiol. 136, 151–175. 10.1016/bs.irn.2017.06.008 29056150

[B41] Takano Y. Machida T. Obara Y. Hirano M. Kudo S. Takagi M. (2014). Methotrexate causes a change in intestinal 5-hydroxytryptamine metabolism in rats. Eur. J. Pharmacol. 740, 496–503. 10.1016/j.ejphar.2014.06.038 24975094

[B42] Takeda N. Hasegawa S. Morita M. Matsunaga T. (1993). *Pica* in rats is analogous to emesis: an animal model in emesis research. Pharmacol. Biochem. Behav. 45, 817–821. 10.1016/0091-3057(93)90126-e 8415820

[B43] Takeda N. Hasegawa S. Morita M. Horii A. Uno A. Yamatodani A. (1995). Neuropharmacological mechanisms of emesis. II. Effects of antiemetic drugs on cisplatin-induced pica in rats. Methods Find. Exp. Clin. Pharmacol. 17, 647–652. 9053584

[B44] Tu Y. (2016). Artemisinin-A gift from traditional Chinese medicine to the world (Nobel lecture). Angew. Chem. Int. Ed. 55, 10210–10226. 10.1002/anie.201601967 27488942

[B45] Wang C. Z. Basila D. Aung H. H. Mehendale S. R. Chang W. T. McEntee E. (2005). Effects of ganoderma lucidum extract on chemotherapy-induced nausea and vomiting in a rat model. Am. J. Chin. Med. 33, 807–815. 10.1142/S0192415X05003429 16265993

[B46] Wang J. Xu C. Liao F. L. Jiang T. Krishna S. Tu Y. (2019). A temporizing solution to “artemisinin resistance”. N. Engl. J. Med. 380, 2087–2089. 10.1056/nejmp1901233 31018065

[B47] WHO (2006). Guidelines for the treatment of malaria. 2nd Edn. Geneva, Switzerland: World Health Organization.

[B48] WHO (2018). World malaria report 2018 *.* Geneva, Switzerland: World Health Organization.

[B49] Wojnicz A. Avendaño Ortiz J. Casas A. I. Freitas A. E. G. López M. Ruiz-Nuño A. (2016). Simultaneous determination of 8 neurotransmitters and their metabolite levels in rat brain using liquid chromatography in tandem with mass spectrometry: application to the murine Nrf2 model of depression. Clin. Chim. Acta 453, 174–181. 10.1016/j.cca.2015.12.023 26712273

[B50] Wynn R. L. Essien E. Thut P. D. (1993). The effects of different antiemetic agents on morphine-induced emesis in ferrets. Eur. J. Pharmacol. 241, 47–54. 10.1016/0014-2999(93)90931-7 8223924

[B51] Yamamoto K. Takeda N. Yamatodani A. (2002). Establishment of an animal model for radiation-induced vomiting in rats using pica. J. Radiat. Res. 43, 135–141. 10.1269/jrr.43.135 12238327

[B52] Yamamoto K. Nakai M. Nohara K. Yamatodani A. (2007). The anti-cancer drug-induced pica in rats is related to their clinical emetogenic potential. Eur. J. Pharmacol. 554, 34–39. 10.1016/j.ejphar.2006.09.058 17109847

[B53] Yamamoto K. Asano K. Matsukawa N. Imaizumi M. Yamatodani A. (2011). Time-course analysis of pica in rats using an automatic feeding monitoring system. J. Pharmacol. Toxicol. Methods 63, 30–34. 10.1016/j.vascn.2010.04.011 20451632

[B54] Yamamoto K. Asano K. Tasaka A. Ogura Y. Kim S. Ito Y. (2014). Involvement of substance P in the development of cisplatin-induced acute and delayed pica in rats. Br. J. Pharmacol. 171, 2888–2899. 10.1111/bph.12629 24641692 PMC4243862

[B55] Zhong W. Chebolu S. Darmani N. A. (2016). Thapsigargin-induced activation of Ca2+-CaMKII-ERK in brainstem contributes to substance P release and induction of emesis in the least shrew. Neuropharmacology, 103, 195–210. 10.1016/j.neuropharm.2015.11.023 26631534

[B56] Zhou T. C. Jang C. S. (1943). Research on the domestic antimalarial drug changshan. (1) changshan (preliminary report). Natl. Med. J. India. (Chongqing Edition) 29, 137–142.

[B57] Zhu S. Zhang Q. Gudise C. Wei L. Smith E. Zeng Y. (2009). Synthesis and biological evaluation of febrifugine analogues as potential antimalarial agents. Bioorg. Med. Chem. 17, 4496–4502. 10.1016/j.bmc.2009.05.011 19467876 PMC2746662

